# Integrated bite case management within a One Health framework for rabies elimination at the primary care level in Kerala, India: an implementation research protocol using a stepped-wedge cluster randomised design

**DOI:** 10.1136/bmjopen-2026-117497

**Published:** 2026-09-23

**Authors:** Zinia Thajudeen Nujum, Martha M Luka, Dhanya Raghunathan, Mufeeda Beegum, Harikumar Sivanandan, Prapti Bajaj, Balaji Chandrashekar, Rajamohanan K Pillai, Thomas Mathew, Biju Soman, Miqdad Asaria, Reka Karuppusami, Andrew Gibson, Manjula Radhakrishnan Sreenath, Arun Jose, Rony Ray John, Sujatha Aparna, Prathapan Rema Prathiush, Swapna Susan Abraham, Sajeev Beena Narayanan, Ummuselma Chungath, Pillaveetil Sathyadas Indu, Anuja Ushakumari, Remadevi Sivaram, Vijayakumar Krishnapillai, Suevia Sudheer Aishwarya, Katie Hampson

**Affiliations:** 1 School of Public Health, Kerala University of Health Sciences, Thiruvananthapuram, Kerala, India; 2 Department of Community Medicine, Government Medical College Kollam, Kollam, Kerala, India; 3 School of Biodiversity, One Health and Veterinary Medicine, College of Medical, Veterinary and Life Sciences, University of Glasgow, Glasgow, UK; 4 Public Health dentistry, PMS College of Dental Science and Research, Thiruvananthapuram, Kerala, India; 5 Mission Rabies India, Thiruvananthapuram, Kerala, India; 6 Directorate of Health Services, Thiruvananthapuram, Kerala, India; 7 Compassion for Animals Welfare Association, Patiala, Punjab, India; 8 Mission Rabies India, Panaji, Goa, India; 9 Achutha Menon Centre for Health Sciences Studies, Sree Chitra Tirunal Institute for Medical Sciences and Technology, Thiruvananthapuram, Kerala, India; 10 Department of Health Policy, London School of Economics and Political Science, London, UK; 11 Christian Medical College and Hospital Vellore, Vellore, Tamil Nadu, India; 12 Worldwide Veterinary Service, Cranborne, UK; 13 National Health Mission, Thiruvananthapuram, Kerala, India; 14 Directorate of Animal Husbandry, Thiruvananthapuram, Kerala, India; 15 State Institute for Animal Diseases, Thiruvananthapuram, Kerala, India; 16 Kerala Veterinary and Animal Sciences University, Pookode, Kerala, India; 17 Government of Kerala Local Self Government Department, Thiruvananthapuram, Kerala, India; 18 Government Medical College Kollam, Kollam, Kerala, India; 19 Directorate of Medical Education, Thiruvananthapuram, Kerala, India; 20 Centre for Advanced Research Information Network and Guidance, Thiruvananthapuram, Kerala, India; 21 Health Action by People, Thiruvananthapuram, Kerala, India

**Keywords:** Delivery of Health Care, Integrated, Neglected Diseases, Primary Health Care, Implementation Science, India

## Abstract

**Introduction:**

Rabies remains a persistent public health threat in India, while gaps in surveillance and coordination of control measures undermine progress towards ending human rabies deaths. The WHO advocates a One Health approach, combining mass dog vaccination, postexposure prophylaxis (PEP) and Integrated Bite Case Management (IBCM). IBCM is not yet incorporated into India’s national rabies action plan for rabies elimination. This study seeks to generate robust evidence on the effectiveness, cost-effectiveness, feasibility, facilitators and barriers of IBCM implemented within Kerala’s Rabies Control Programme.

**Methods and analysis:**

This implementation research adopts a stepped-wedge cluster randomised controlled trial (RCT) design. IBCM will be implemented across six administrative blocks in Thiruvananthapuram District, Kerala, across diverse rural and urban settings and including tribal population representation. The IBCM intervention includes stakeholder training and support and active animal surveillance aligned with WHO guidance. Suspect or probable rabies exposures presenting at selected health facilities will be identified via a community-based hotline. This will trigger investigations and responses including dog vaccination and sensitisation. Prior to IBCM implementation, baseline data will be collected on management of dog bites, bite patient incidence and healthcare seeking behaviours. Hospital-based event tracking of bite cases will be conducted at enrolled public health facilities before and during IBCM implementation. The resulting RCT data will be used to model the impacts and cost-effectiveness of IBCM, including the potential for cost savings through judicious PEP if scaled across Kerala. To understand the barriers and facilitators to IBCM implementation, we will use in-depth interviews and focus group discussions with stakeholders.

There is an urgent need to strengthen rabies surveillance, including coordination across health and animal health sectors and outbreak responses to accelerate rabies elimination. IBCM is expected to lead to improved PEP completion, increased detection and laboratory confirmation of rabid dogs, strengthened dog vaccination coverage and targeted outbreak responses in high-risk areas. This RCT will provide critical evidence to inform policy, support scale-up, generate insights into contextual barriers and facilitators for IBCM implementation, and guide India’s One Health rabies elimination strategy. This study will evaluate a health-sector-initiated IBCM approach that identifies cases through dog-bite patients presenting to health facilities, supporting comprehensive One Health integration and improved patient care.

**Ethics and dissemination:**

The study was approved by WHO Ethics Committee (2024.9.Ind), Kerala University of Health Sciences (AX04/SOP07A/V2) and the ethics committee of Government Medical College, Thiruvananthapuram (HEC No.12/06/2024/MCT); informed written consent will be obtained from all participants, analysis will be limited to de-identified data, and findings will be disseminated through peer-reviewed publications and policy-relevant platforms.

**Trial registration number:**

CTRI/2024/08/073117.

**STRENGTHS AND LIMITATIONS OF THIS STUDY**

- This study evaluates Integrated Bite Case Management for rabies surveillance implemented within the primary healthcare system, with coordination embedded at the level of primary care delivery.
- The study employs an implementation research approach using a cluster randomised trial with a stepped-wedge design, allowing ethical phased rollout and facilitating replication and scale-up in similar health system settings.
- A major strength is the potential integration of multiple stakeholders from the health sector, animal sector, local self-government and non-governmental organisations to support coordinated One Health implementation.
- The mixed-methods design—combining a cluster randomised trial to assess effectiveness, qualitative process evaluation to identify facilitators and barriers, baseline assessment of problem magnitude and cost-effectiveness analysis using real-world data—enables comprehensive and policy-relevant evidence generation.
- Due to resource constraints, continuous field follow-up is not feasible and monitoring will therefore be conducted on prespecified days rather than on a continuous basis.

## Introduction

 Rabies is a neglected tropical disease endemic in over 150 countries, causing an estimated 59 000 deaths annually and an economic burden of around US$8.6 billion.^1^ Current rabies prevention strategies largely rely on postexposure prophylaxis (PEP), which involves vaccinating individuals following exposure to potentially rabid animals. One Health is a unifying approach that aims to sustainably balance and optimise the health of humans, animals and ecosystems and emphasises integrated interventions across human, animal and environmental sectors.^2 3^ Mass dog vaccination is needed to eliminate dog-mediated rabies;^4^ however, global investment in dog vaccination remains disproportionately low,^1^ while spending on PEP remains high.

Coordinated surveillance between human and animal health sectors is crucial for detection of animal rabies cases and for evaluating the effectiveness of control programmes. This forms the basis of ‘Integrated Bite Case Management’ (IBCM), an advanced One Health surveillance method that connects health professionals, veterinary professionals and the community.^5–10^ Modelling suggests that a combination of mass dog vaccination, improved PEP access and IBCM is the most cost-effective strategy for reducing human rabies deaths.^11^ WHO provides guidance on IBCM,^12^ but implementation research evidence from diverse real-world settings, including on intersectoral engagement, is still lacking.

India bears a disproportionate burden of global rabies deaths. The latest countrywide survey suggests an overall decline to around 6000 rabies deaths per year**,**^13^ attributable to improved public health infrastructure, awareness, human rabies vaccine availability and intradermal vaccination methods in many parts of the country. To further this progress, India has established the National Action Plan for Rabies Elimination (NAPRE), aiming to eliminate dog-mediated rabies deaths by 2030.^14^ While NAPRE focuses on mass dog vaccination and PEP provisioning, it does not explicitly incorporate IBCM as part of its surveillance strategy.^15^

Kerala, India’s most advanced state in terms of Human Development Index, reports around 1.6% of its population sustaining animal bites annually according to the Integrated Disease Surveillance Programme (IDSP) data for 2018–2022 (pers. comm, Department of Public Health, Directorate of Health Services (DHS)). Annual rabies deaths in Kerala range between 5 and 33 since 2018.^16^ The state’s robust primary healthcare system, decentralised governance and active community participation create an ideal environment for implementing One Health strategies like IBCM. As part of the Rebuild Kerala Initiative, a World Bank funded project to support socio-economic development in Kerala, the government launched a Centre for One Health.^17^ Nearly 900 000 people in Kerala receive PEP every year (pers comm, Department of Public Health, DHS), placing a growing financial burden on the government. In this context, introducing IBCM offers an opportunity to improve the efficiency of PEP use by more appropriate targeting, while strengthening the link between human and animal health sectors and improving dog vaccination to address the source of transmission. This integrated One Health approach could make rabies prevention more effective, sustainable and aligned with the state’s One Health vision.

A recent systematic review on the cost-effectiveness of One Health rabies interventions highlighted a scarcity of high-quality implementation studies.^18^ This study is designed to fill that evidence gap, by carrying out a stepped-wedge cluster randomised controlled trial (RCT) of IBCM implementation in Kerala. It aims to examine the effectiveness of IBCM for improving rabies prevention, control and surveillance, in terms of PEP completion among bite victims, detection of animal rabies cases and enhanced dog vaccination. We further aim to undertake cost-effectiveness modelling and identify the facilitators and barriers influencing the implementation of IBCM, via implementation outcomes of acceptability, adoption, appropriateness, feasibility, fidelity, penetration and sustainability.

## Methods and analysis

### Study design

We will use a pragmatic, stepped-wedge cluster RCT to evaluate the implementation of IBCM in Kerala, India. The study will involve six clusters and will span a pre-implementation period (Preparatory Phase) prior to the stepped rollout of IBCM (Implementation Phase) followed by a period of sustained implementation (Sustenance Phase); (figure 1). Data collection will continue throughout. Baseline data collected prior to IBCM implementation will capture existing practices in the management of dog bite cases in the absence of IBCM. A cross-sectional survey (online supplemental file 1) will assess the incidence of animal bites and related healthcare-seeking behaviour to inform analyses. The sequential rollout of IBCM across clusters will be determined through randomised allocation. An embedded process evaluation will explore the acceptability, adoption, appropriateness, feasibility, fidelity, penetration and sustainability of IBCM, guided by the Reach, Effectiveness, Adoption, Implementation and Maintenance (RE-AIM) framework.^19^ The measured outcomes will be used to model the impacts and cost-effectiveness of IBCM if scaled up across the state of Kerala.

**Figure 1:**
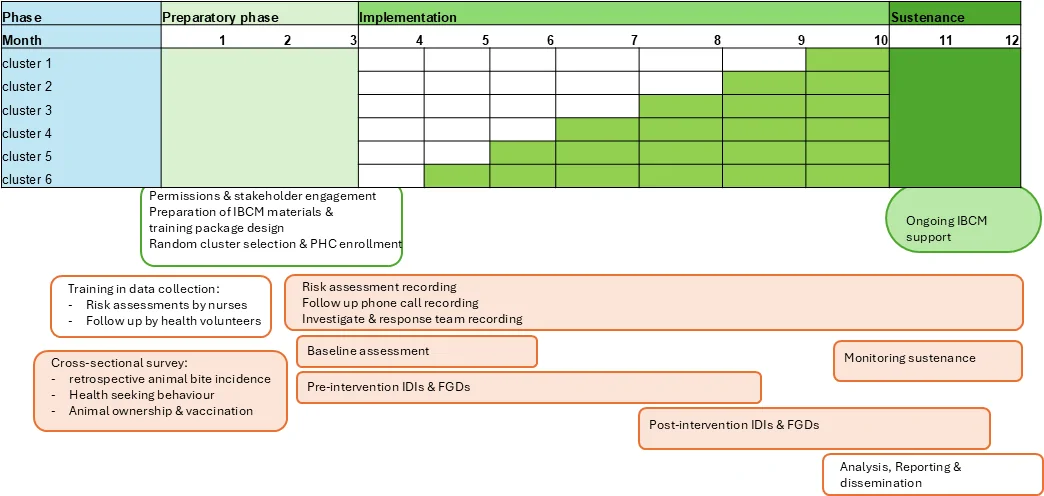
Study design for stepped-wedge cluster randomised controlled trial of IBCM in Kerala, India. FGDs, focus group discussions; IBCM, Integrated Bite Case Management; IDIs, in-depth interviews; PHC, primary health centre.

### Study setting and population

The study is conducted in Thiruvananthapuram district, Kerala, India (figure 2). Kerala comprises 14 districts, among which Thiruvananthapuram consistently reports the highest burden of dog bites. State-level data from the IDSP shows an annual incidence of 15 animal bite patients and 8.3 dog bite patients seeking hospital care per 1000 population in Thiruvananthapuram, almost all of whom receive PEP (pers. comm, Department of Public Health, DHS).

**Figure 2:**
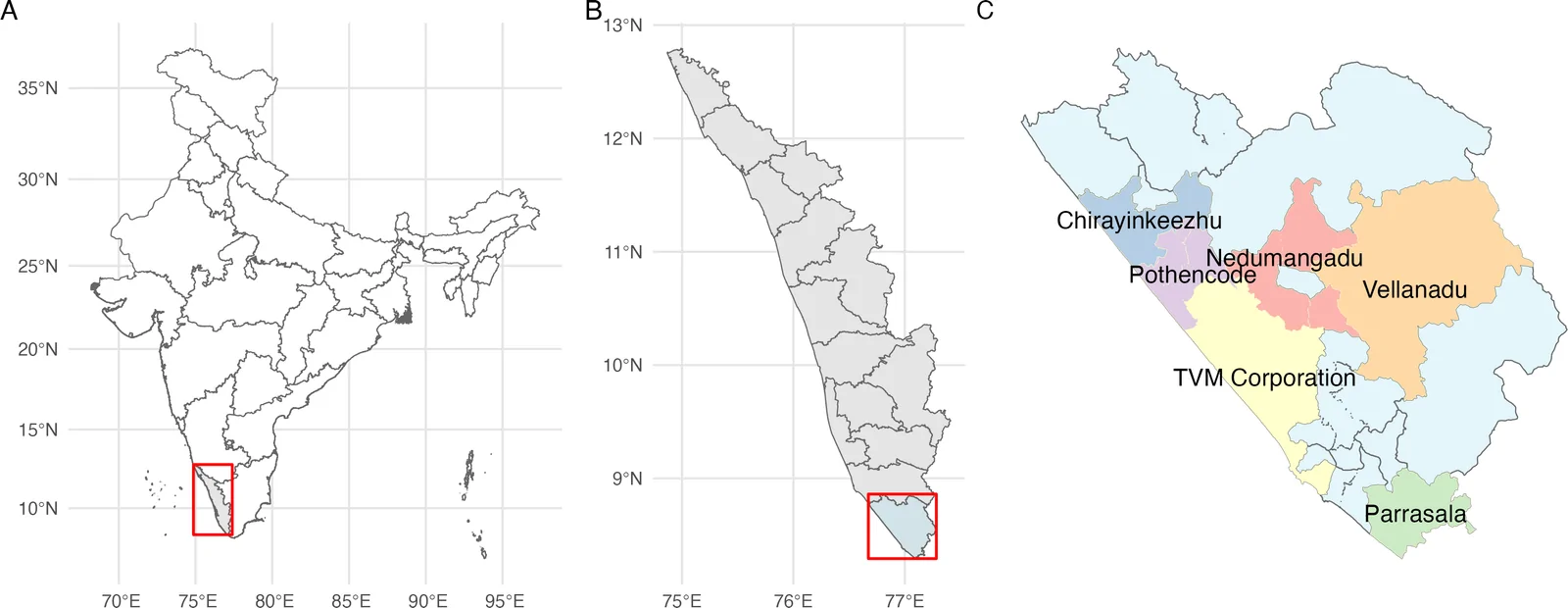
Map of the study setting: (**A**) India showing Kerala; (**B**) Kerala showing Thiruvananthapuram district; and (**C**) Thiruvananthapuram district showing the six clusters selected for IBCM implementation, Kerala, India. IBCM, Integrated Bite Case Management.

Primary healthcare in the district is delivered through primary health centres (PHCs),^20^ some of which have been upgraded to family health centres (FHCs).^21^ Typically, five to six PHCs/FHCs are grouped under a block PHC or a community health centre.^20^ Thiruvananthapuram district comprises 11 rural blocks and three urban administrative zones.^22^ We will select six clusters (table 1), with each defined as an administrative block/area (a subdistrict unit of governance in India). These are stratified to include five rural blocks, including one with a sizeable tribal population purposively selected to ensure adequate representation of tribal communities and one urban administrative zone (Thiruvananthapuram corporation).

**Table 1:** Scoring to select study sites based on animal and human rabies data and dog bite cases

Sl. no.	Site	Lab confirmed animal rabies reported (2022)	Lab confirmed animal rabies reported (2023)	Human rabies deaths 2022 and 2023	No of times the area figured in the top 10 areas based on dog bite cases/month (January–August 2022)	Total score*	Rank
1	Parassala	0	2	1	3	6	4
2	Perumkadavila	1	0	0	1	2	0
3	Athiyannoor	0	1	0	1	2	0
4	Nemom	0	0	0	0	0	0
5	Pothencode	3	0	1	4	8	3
6	Vellanad†	0	0	0	0	0	0
7	Nedumangad	1	3	1	5	10	2
8	Vamanapuram	0	1	1	2	4	0
9	Kilimanoor	0	1	1	2	4	0
10	Chirayinkeezhu	3	0	0	3	6	5
11	Varkala	1	0	1	2	4	0
12	Thiruvananthapuram Corporation	1	5	3	9	18	1
13	Nedumangad Municipality	2	0	0	2	4	0
14	Attingal Municipality	0	1	1	2	4	0

* Using data from three sources namely lab-confirmed animal rabies cases in 2022 and 2023 (from State Institute of Animal Diseases), human cases reported in 2022 and 2023 and reported dog bite cases/month reported to veterinary hospitals/dispensaries (January to August 2022—if the area was listed in the top 10 areas according to dog bite cases and if the numbers were above 15 cases/month it was given a score of 1, each month that it appeared considered) a scoring system was developed. Each time a block was represented in the data a score of 1 was given.

† Purposively included sixth cluster for tribal representation.

The study participants will be the patients presenting to PHCs with dog bite(s)/scratch(es)/other potentially rabies-risk contacts with dogs (defined as a lick to abraded skin or mucosal membrane or substantive contact with a dog identified as rabid) and administered PEP. Exposures from other animals will be excluded. For pragmatic purposes, the first two dog bite patients presenting each day to the enrolled PHCs will be recruited to the IBCM intervention. To assess the reach and coverage of the surveillance system, the total number of dog-bite patients presenting to each participating PHC during the study period will be recorded and reported alongside the number enrolled for detailed follow-up.

For the process evaluation, study participants will include purposively selected stakeholders involved in IBCM including political leaders at state, district and local levels, programme managers from the human and animal health sectors, medical officers and health workers from both the DHS and Animal Husbandry Department (AHD), Accredited Social Health Activists (ASHA) workers and individuals exposed to animal bites for beneficiary case studies.

### The IBCM intervention

#### Overview

We developed an IBCM protocol based on WHO’s Open Course Module 8 on IBCM^12^ making adaptations for implementation in Kerala, so that the IBCM risk assessment is not used for PEP decision-making. The intervention will engage representatives from the DHS and the Directorate of Medical Education, the AHD, local self-government bodies, community health workers and animal health stakeholders from the non-governmental organisations (NGOs) Mission Rabies and Compassion for Animal Welfare Association, ensuring a multisectoral approach aligned with One Health principles.

We will use the following case definitions^12 23^: a *suspect* rabid animal must show at least one A—Aggression, L—lethargy, P—Paralysis, H—Hypersalivation and A—Abnormal Vocalisation (ALPHA) sign at the time of or within 10 days of the exposure; a *probable* rabid animal is a suspect animal with additional history of a bite by another suspect/probable animal and/or is killed/died/disappears within 4–5 days of showing signs. A *confirmed* rabid animal is a rabies suspect/probable animal that is confirmed in the laboratory. An animal is defined as not a case if it survives a 10-day observation, or is a suspect/probable case ruled out by laboratory testing.

#### Intervention components

The IBCM intervention comprises *notification*, *investigation* and *response* steps as illustrated in figure 3.

**Figure 3:**
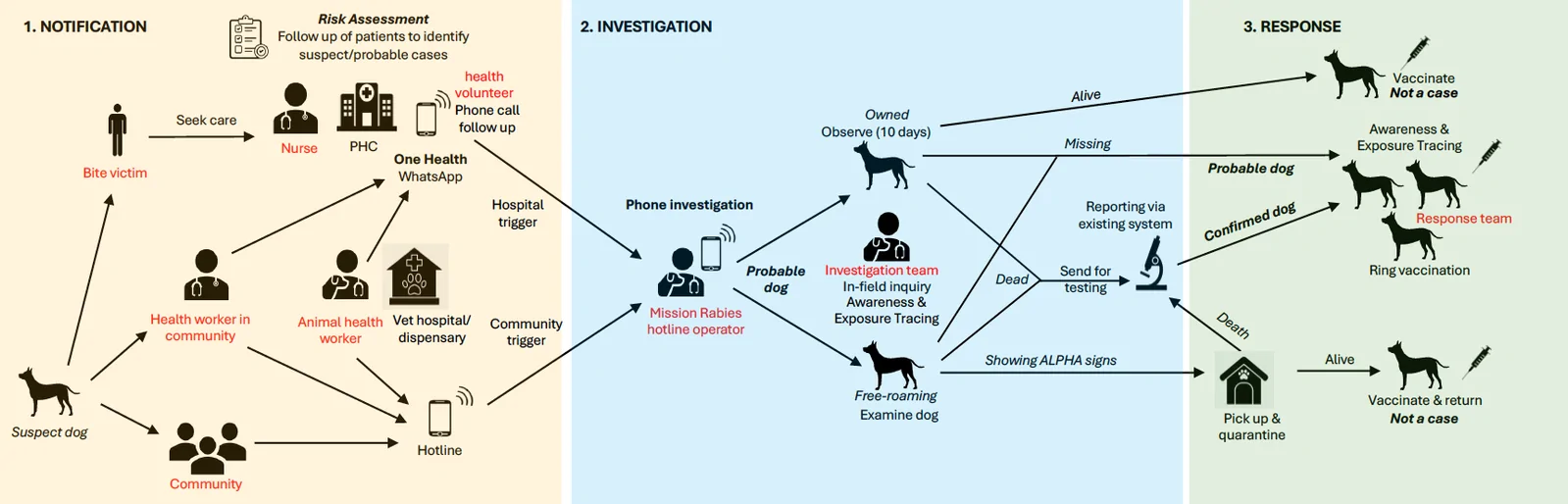
Schematic of IBCM intervention to be implemented for the RCT*.* Health workers in the community are ASHA workers, or junior public health nurses or junior health inspectors; animal health workers are veterinary doctors or livestock inspectors. PHCs are primary health centres. LSGD are local self government departments, HD are health departments, AHD are animal Health departments. Investigation and response teams comprise Mission Rabies and CAWA staff with support from the LSGD, HD and AHD as required. ALPHA, Aggression, Lethargy, Paralysis, Hypersalivation and Abnormal Vocalisation; CAWA, Compassion for Animal Welfare Association; IBCM, Integrated Bite Case Management; RCT, randomised controlled trial. People are indicated in red, and outcomes in bold italic.

#### Notification

The notification step involves triggers for investigations via two routes: from PHCs (hospital trigger event) and from communities (community trigger event). Hospital trigger events will be a suspected, probable or confirmed rabies exposure reported to a PHC. Community trigger events will be reports from a community when a dog bites more than one person or animal, that is, a cluster of bites or any dog reported as suspect, probable or confirmed rabies, via a free public Mission Rabies hotline. Any member of the community or health or animal health professionals can report via the hotline. These notifications come to a coordinated One Health WhatsApp group involving stakeholders from health, animal and NGO sectors for veterinary, health personnel or community members to report suspect rabid animals (figure 3).

At the PHC patients’ will first be assessed by the designated medical officer to determine their PEP requirements, based on the 2019 National Guidelines^24^ and a nurse will administer PEP according to the standard of care. The nurse will then undertake an IBCM risk assessment by asking the bite patient questions about the biting dog adapted from WHO guidance.^25^ Volunteer health workers will subsequently call these patients over the phone on days 0, 10 and 28 to encourage completion of the intradermal rabies vaccination schedule and follow-up on the health and vaccination status of the dog.

The day 0 call is used to identify additional individuals bitten by the same dog and the bite victim is requested to encourage these others to seek care. If there are multiple bites in the community by the suspect/probable dog these events are passed on to the response team via the One Health WhatsApp group (hospital trigger, figure 3). During the call patients will be made aware of ALPHA signs to identify rabies, and will be asked whether the dog is alive and observable or has died, been killed or is missing. For dogs that are observable, patients are requested to monitor the dog at home for 10 days and immediately report via the hotline if any ALPHA signs appear. If the dog is suspect or probable for rabies, meaning the dog has died, been killed or disappeared, or ALPHA signs are reported during the observation period, a response is initiated. The day 10 follow-up call will check if the dog remains alive and if it is unvaccinated, owners will be urged to vaccinate the dog at the nearest veterinary hospital. The day 28 follow-up call will determine whether the patient has completed their PEP schedule and, if applicable, whether dog vaccination was initiated.

#### Investigation

Following a hospital or community trigger, the hotline operator will enquire to clarify the dog’s health and confirm whether and how an in-field inquiry should take place. If the call confirms the need for an in-field inquiry, a response team will travel to the site and examine the dog. If it is already dead the animal is sent for laboratory testing. If the animal is alive but owned, the owner is requested to observe the animal under home quarantine to ensure it remains healthy, or call the response team if it shows ALPHA signs or dies. During the investigation community sensitisation is undertaken, including distribution of awareness materials and any other in-contact individuals are identified and counselled.

#### Response

For any dog identified as probable or confirmed as rabid, a response will be undertaken. Awareness sessions will be done door-to-door and in group sessions within communities including distribution of materials (pamphlets and hotline cards). Ring vaccination of all dogs in the radius of one kilometre around the site of the identified rabid dog will be undertaken with the guidance of the Worldwide Veterinary Services (WVS) app.^26 27^ If the dog is alive and free-roaming but showing ALPHA signs, the response team will pick up the dog to take it to the isolation facility for monitoring. If the dog dies, it will be sent to the laboratory for testing. If the dog remains alive and healthy for 10 days, it will be vaccinated and returned to the community.

### IBCM implementation

Detailed roles and responsibilities of stakeholders implementing IBCM are outlined in table 2. At the start of the intervention in each cluster, relevant stakeholders including both health and veterinary personnel will undergo joint training on IBCM, followed by ongoing support for implementation, including provisions for surveillance tools, testing and reporting systems. A package of awareness materials will be distributed to reinforce messages on dog bite prevention, rabies awareness and the importance of vaccination of both bite victims and dogs. At the trainings, all participants (staff from the enrolled PHCs, veterinary hospitals or dispensaries, and attending community members such as ASHAs and community leaders, etc) will be requested to add the hotline number to their phone and will be oriented to report community trigger events to the hotline number (figure 3). A community awareness programme will also be implemented in each cluster at the onset of the intervention. This includes sensitisation of key stakeholders such as local self government department (LSGD) representatives, and repeat sessions will be conducted as necessary.

**Table 2:** Responsibilities of stakeholders for IBCM implementation and the overall study

Stakeholder	Responsibilities
Directorate of Health Services State and district programme officers	Leaders of the state and district rabies control programme. All communications to the primary care level (PHC/FHC) will be channelled through them
Medical officers of PHC	Implementation at the primary healthcare level Wound categorisation based on routine risk assessment (based on national protocol) Management of the bite cases Coordination with animal health personnel
Nursing officer at PHC	Administers WHO risk assessment (including animal history and status) Reporting of the two events to the WhatsApp group daily for follow-up by the volunteers Detailed data collection on two events in ODK form
Health workers, ASHA	Community education, monitoring and reporting of events in households, work in liaison with project field staff
Project field staff including paid volunteers	Documentation, data entry, monitoring and surveillance, linkage between the human and animal health personnel
Directorate of Animal Husbandry	Leaders of the state and district rabies control programme. All communications to the primary care level (government veterinary hospital medical officer and team) will be channelised through them
State Institute of Animal Diseases	Investigations—Lab tests antibody titres postvaccination and testing of rabies in suspect animals, quality control of sample collection, storage, transportation and testing, timely reporting of results
Indian Veterinary Association, Kerala	Mobilising support at the field level from veterinary personnel, advocacy and training
Government Veterinary Hospital medical officers	Assessment and monitoring of the health status of the dog—initial and when required, communication with the PHC medical officer, supportive supervision of the field staff and livestock inspectors
Livestock inspectors	Reporting of hospital or community trigger events
Global collaborator from the University of Glasgow	Technical support in implementation, training and analysis
Mission Rabies and Compassion for Animal Welfare Association	Technical support for implementation, training in IBCM, animal surveillance and response, pick up, assessment and monitoring of health status of free-roaming dogs during quarantine/observation, sample collection for testing suspect free-roaming dog, ring vaccination around probable and confirmed rabies dogs
Local self government—political leaders, officials in the block panchayat	Community engagement, advocacy, ensuring sustainability, social audit
Co-investigator from Achutha Menon Centre for Health Science Studies, Kerala	Support with the ODK data entry form development, storage and maintenance of data
Principal investigator and team at School of Public Health, Kerala University of Health Science, Department of Medical Education—Government Medical College, Kollam	Overall project planning, coordination and management (technical and financial), submission of reports periodically, documentation and convening of meetings with stakeholders Conduct of workshops, development of software for data entry, engaging students posted in the community trainings, dissemination workshops and policy briefs, incorporation into curriculum of medical and paramedical students

ASHA, Accredited Social Health Activists; FHC, family health centre; IBCM, Integrated Bite Case Management; ODK, Open Data Kit; PHC, primary health centre.

A One Health WhatsApp group will be set up for each cluster at the start of the study, enrolling relevant stakeholders from PHCs and the LSGDs. Throughout the study the enrolled nurse at each PHC will enter minimal trigger details for the two patients recruited daily into the One Health WhatsApp group.

During the sustenance phase, the implementation of IBCM will be monitored across all clusters. Ongoing support and technical guidance will be provided as needed to ensure continuity and fidelity to the intervention.

#### Outcomes

The study is guided by the RE-AIM framework (table 3). *Effectiveness* will be measured quantitatively, through changes in PEP completion, dog health status, surveillance and dog vaccination indicators across intervention and control cluster periods. *Reach* will be examined through trigger events. *Adoption* will be assessed at the setting and staff levels, capturing participation rates. As part of adoption, client satisfaction will be assessed using structured questions integrated into the evaluation tool. *Implementation* will be assessed based on the degree of fidelity to IBCM components and adaptations undertaken, including consistency across contexts. Finally, *Maintenance* will be evaluated at the individual and setting levels through follow-up of health system performance indicators beyond the intervention period over the sustenance phase, focusing on continued service delivery and adherence to the implementation protocol. These indicators will be monitored through weekly IDSP review meetings, which are regularly conducted with doctors and healthcare workers at each PHC. All the dimensions of the RE-AIM framework shall also be captured qualitatively.

**Table 3:** RE-AIM framework used to evaluate IBCM implementation

RE-AIM dimension	Outcomes	Indicator
Reach	Trigger event reach	Percentage of events reported during study period that were enrolled and followed up
Effectiveness	Patient	PEP completion percentage
		Patient satisfaction score
	Dog health	Quarantine
		Shown to a veterinary doctor
		Satisfaction with the care of animals score
		Perceived animal welfare score
	Surveillance	Dog health status (confirmed, probable or suspect rabies or healthy)
	Response	Number of tested dogs
		Proportion of dogs that test positive for rabies
		Rapid responses with ring vaccination completed
		Owned dog vaccination initiated in healthy (unvaccinated) dogs post-observation
Adoption	Uptake	Percentage of settings that participate out of those approached
		Percentage of staff invited that willingly participate
Implementation	Fidelity and adaptation	Percentage of IBCM components implemented well
		Adaptations made to IBCM during the study
		Consistency of implementation across staff/settings/subgroups (process indicators)
Maintenance	Post implementation	Number of calls received by the hotline 3 months after implementation versus during intervention phase
		Effectiveness indicators measured 3 months after the implementation phase overall and at cluster level

IBCM, Integrated Bite Case Management; PEP, postexposure prophylaxis.

The study shall also include a structured implementation fidelity monitoring plan. Core intervention components (WhatsApp communication pathways, hotline functionality, referral linkages and response coordination) will be tracked using predefined process indicators across clusters and over time. Routine monitoring will document whether each component is functional and actively used, and implementation performance will be reviewed periodically to identify gaps and support corrective actions where required. This will allow assessment of both intervention effectiveness and consistency of delivery across sites.

The implementation phase of the study commenced on first of March 2025 with an original intended primary completion date of the sustenance phase till February 2027 to allow for adaptation to routine working.

### Data collection and analysis

Risk assessment data for the two bite patients recruited daily to the study at each enrolled PHC will be recorded by nurses. Data on each of the follow-up calls (day 0, 10 and 28) to bite patients will be collected by the health volunteer. The risk assessment (online supplemental file 2) and phone call follow-up (online supplemental file 3) collection tools will be converted into electronic forms using the Open Data Kit platform. Data on the investigation and response will be collected by the investigation and response teams in the WVS app.

A generalised linear mixed model (GLMM) will be used for analysis, with fixed effects for intervention exposure and time periods to account for secular trends inherent in the stepped-wedge cluster design. The model will include random effects for clusters (PHCs) to account for within-cluster correlation. This structure enables estimation of intervention effects while appropriately adjusting for both clustering and temporal confounding. Subgroup analysis will be done across clusters and urban–rural settings. We hypothesise that PEP completion rates of recruited patients will increase under IBCM, and that confirmation and vaccination of otherwise unvaccinated healthy dogs will increase, as will detection of probable rabid dogs and laboratory confirmation of cases. We expect that rapid responses and associated ring vaccination following case detection will also increase with IBCM. Data from the risk assessment will be used to calculate the potential for cost savings through judicious PEP. Answers to the risk assessment questions will be scored and computed to identify whether the individual required PEP as per the WHO risk assessment. The proportion of PEP that could have been avoided will be used in the cost-effectiveness analysis.

The costing will include both direct costs, such as PEP, diagnostics and logistics, and indirect costs, including productivity losses and time costs. Data will be obtained from government records, hospital datasets, stock inventories and expert consultations, with annualisation applied to reflect typical implementation costs.

Qualitative data collection will involve in-depth interviews (IDIs) with political leaders at the state (1), district (1) and local (6) levels, programme managers from both the human and animal health sectors (one each at state and district levels), medical officers and healthcare workers from both the DHS and the AHD, ASHA workers and IBCM investigators, as well as individuals exposed to animal bites for beneficiary case studies. Data collection will be guided by semistructured IDI and focus group discussion (FGD) guides (online supplemental file 4 and online supplemental file 5), developed to capture key implementation outcomes. IDIs and FGDs will be scheduled at the convenience of the participants, with prior appointments and permissions and will be conducted in settings that ensure privacy and comfort for participants. All interviews and FGDs will be recorded with prior consent, with 5% of sessions directly supervised.

Recordings of IDIs and FGDs will be transcribed verbatim and translated into English when required, with 5% of transcripts cross-verified against recordings, with replacements made when necessary. Thematic analysis will be undertaken using both manual methods and NVivo software to ensure systematic organisation and rigour. Responses will be grouped to identify domains of homogeneity across different stakeholder categories, facilitating comparative analysis. Coding and categorisation will be applied to each domain and representative quotations highlighted for depth. Results will be synthesised into an ethnographic summary that integrates thematic patterns with quotable narratives, to provide nuanced insights into stakeholder experiences and perceptions.

A cost-effectiveness analysis will be conducted to evaluate the IBCM intervention compared with the status quo. The primary outcomes will include human rabies deaths averted, corresponding disability-adjusted life years averted, animal rabies cases detected, cost per animal rabies case confirmed and cost savings attributable to judicious use of PEP (through application of the WHO rabies risk assessment framework).

Effectiveness inputs will be derived from the stepped-wedge RCT and incorporated into a stochastic rabies transmission model linked to a probabilistic decision-tree model to capture human and animal health pathways. Model parameters will be informed by published literature, routine surveillance data and findings from the primary study. Two scenarios will be modelled. The status quo scenario will assume provision of PEP without systematic application of the WHO risk assessment chart and continuation of current levels of mass dog vaccination. The One Health IBCM scenario will assume systematic implementation of the WHO risk assessment chart, integration of IBCM between human and animal health sectors, and continuation of mass dog vaccination.

Incremental cost-effectiveness ratios will be calculated and compared against relevant country-specific cost-effectiveness thresholds. Parameter uncertainty will be explored using one-way sensitivity analyses and probabilistic sensitivity analysis employing Monte Carlo simulation.

The base-case analysis will adopt a 5-year time horizon (2025–2030) to capture medium-term outcomes of IBCM scale-up, with an additional 10-year horizon modelled to assess longer-term impacts. Both costs and health outcomes will be discounted at an annual rate of 3%. Analyses will be conducted from both the healthcare system and societal perspectives. Costs will be reported in Indian rupees and converted to US dollars using the exchange rate prevailing at the time of project initiation.

The overall study design is summarised in table 4

**Table 4:** Study design for data collection and analysis to determine whether IBCM achieved the desired outcomes

Objective	Criteria (how we know we have achieved the objectives) and information required	Data collection tools	Procedures	Study population	Analyses
To evaluate the effectiveness of IBCM for rabies prevention, control and surveillance, in terms of PEP completion, enhanced dog vaccination and detection of rabies cases.	Percentage change in PEP completion with IBCM implementation	ODK follow-up form	Phone call follow-up	Patients who are recruited from the PHC	Generalised linear mixed-effects model to account for clustering and time.
	Percentage change in the number of dogs seen by the veterinary doctor with IBCM implementation	ODK follow-up form	Follow-up phone call by health worker volunteer on days 0, 10 and 28	Dogs involved in the event among recruited patients	
	Change in percentage of owned dogs vaccinated after the biting event with IBCM implementation	ODK follow-up form	Follow-up phone call by health worker volunteer on day 28	Dogs involved in the event among recruited patients	
	Percentage change in confirmed dog rabies cases with IBCM implementation	WVS app	Phone investigations of trigger events, field inquiry, quarantine observation and testing if required	Dogs involved in the event among recruited patients	
	Number of dogs vaccinated during ring vaccination around probable or confirmed cases	WVS app	Conduct of ring vaccination of dogs using the WVS app to locate and mark the area around probable and confirmed cases and documentation in the app	Dogs identified in the marked area	
To find the cost-effectiveness of IBCM implementation in Kerala.	The cost-effectiveness of IBCM will be modelled, comparing human rabies deaths and animal rabies cases, corresponding DALYs averted, animal case detection, cost per case confirmed and cost reductions by use of the WHO risk assessment.	Outcomes will be derived from the RCT data and modelled using a stochastic transmission model linked to a probabilistic decision-tree model.	Cost of IBCM implementation, potential reduction in cost by using the WHO risk assessment for PEP and effectiveness outcomes will be obtained from the RCT	Population of Kerala over a time horizon of 5 years and 10 years for medium and long term outcomes respectively for scale-up of IBCM	Cost-effectiveness modelling. Two scenarios will be examined: the status quo versus a One Health IBCM approach. Incremental cost-effectiveness ratios will be calculated and compared against the country threshold.
To identify the facilitators and barriers influencing the implementation of IBCM, via implementation outcomes of acceptability, adoption, appropriateness, feasibility, fidelity, penetration and sustainability.	IBCM acceptability, adoption, appropriateness, feasibility, fidelity, penetration, sustainability	In-depth interview and focus group discussion guides	Purposive selection of stakeholders from the six clusters before and after the implementation	Political leaders, programme managers, medical officers and healthcare workers from both the health and animal husbandry and ASHA workers	Thematic analysis to identify barriers and facilitators.

ASHA, Accredited Social Health Activists; DALYs, disability-adjusted life years; IBCM, Integrated Bite Case Management; ODK, Open Data Kit; PEP, postexposure prophylaxis; PHC, primary health centre; RCT, randomised controlled trial; WVS, Worldwide Veterinary Services.

### Sample size

The sample size for the baseline cross-sectional survey is based on national (1.96%) and local (Thiruvananthapuram: 1.5%) estimates of the annual incidence of animal bites. Using the formula for calculating a proportion with 95% confidence (4pq/L^2^); taking p as 0.015 (annual incidence estimate of animal bites), q as (1-p) and L (precision) as an absolute 2; the required sample size is 148, which, when adjusted for a design effect of 2, becomes 296. Assuming 15% for non-response increases the total sample size to approximately 350 individuals. Given an average household size of 4, a total of 88 households will be surveyed. To ensure adequacy, we propose surveying 100 households per cluster and therefore 600 households in the six clusters together.

We estimated the RCT sample size to be sufficient to detect a 15% decline in potential PEP requirements after IBCM implementation among those seeking care, using Pocock’s formula^22^ for comparison of proportions and incorporating a design effect. The expected proportion of events in the control arm was set at 0.75, and in the intervention arm at 0.90, corresponding to a ratio of 0.833.


$$\left[\frac{\left(P_{1}Q_{1}+P_{2}Q_{2}\right)}{\left(P_{1}-P_{2}\right)}\right]\times f(\alpha ,\beta )$$

Where: $P_{1}=0.90$ and is the proportion expected to start PEP prior to IBCM, while $P_{2}=0.75$ corresponds to a reduced proportion requiring PEP under IBCM. $Q_{1}=1-P_{1}=0.10$; $Q_{2}=1-P_{2}=0.25$ and f(α,β)=16, derived from 2×8 for a significance level of α=0.05 and power 1-β=0.80. We assume a design effect of 1.95, DE=1+(number in each cluster-1) × intraclass correlation (ICC); based on an average cluster size of 20 and an ICC of 0.05. Thus, the required sample size per arm is 385 trigger events, which is feasible considering the anticipated number of events (n=420).

For the process evaluation, a stratified purposive sampling technique will be used to ensure representation across stakeholder categories and performance levels (eg, best-performing and least-performing ASHA workers). A total of 58 IDIs will be conducted across the six study clusters (table 5). Six FGDs with practising health workers will be conducted before and after IBCM implementation, one in each cluster during both time periods, yielding a total of 12 FGDs. Two beneficiaries per cluster (one before and one after IBCM implementation), preferably dog owners with bite exposure experience, will be selected for detailed case studies and experience sharing.

**Table 5:** Number of In-depth interviews with stakeholders

Sl. no.	Category	Numbers
1	Political leaders	8
2	Programme managers	2
3	Medical officers—Health Department	6
4	Medical officers—Animal Husbandry Department	6
5	Healthcare workers from Health Department	6
6	Healthcare workers from Animal Husbandry Department	6
7	ASHA*	12
8	Beneficiaries	12
	TOTAL	58

* ASHA—voluntary workers selected from the community working for the health.

ASHA, Accredited Social Health Activist.

### Patient and public involvement statement

Patients and members of the public will be actively involved in multiple stages of this implementation research on IBCM. During the study design phase, patients and community members will participate in qualitative interviews to inform the development of intervention strategies and ensure contextual relevance. Community members will also be engaged in ongoing surveillance activities, including the reporting of clusters of animal bite events and the identification and notification of suspected or probable rabies cases in dogs.

Public participation will further extend to implementation activities such as ring vaccination of dogs and community-based education and awareness programmes on rabies prevention and appropriate health-seeking behaviour following animal bites. Local political leaders and community representatives will be involved throughout the design, implementation and monitoring phases to facilitate community engagement, coordination across sectors and local ownership of the intervention.

As part of the dissemination process, study findings and outcomes will be shared with participating communities and stakeholders to communicate the effectiveness of the intervention and to support learning, accountability and sustainability

## Discussion

IBCM is increasingly recognised as a valuable One Health approach, fostering collaboration between human health and veterinary services to ensure timely detection, reporting and response to suspected rabies exposures. In Kerala, where human–dog interactions are frequent and rabies remains endemic,^28^ IBCM can serve as a bridge linking bite patient management with animal surveillance. By strengthening veterinary reporting and laboratory confirmation of rabies in animals, the approach can guide immediate public health responses while informing targeted dog vaccination campaigns, thereby addressing the root source of transmission and advancing progress towards the state’s 2030 rabies elimination goals.

A distinctive feature of our protocol is the adaptation of IBCM to the local policy and stakeholder context. While WHO’s IBCM guidance emphasises rationalised use of PEP by linking administration to animal rabies investigation outcomes, this component is not prioritised in our study. Stakeholder consultations revealed reluctance to reduce PEP administration, given divergence from current national rabies prevention guidelines, which emphasise universal PEP coverage. Accordingly, our IBCM approach focuses on strengthening animal surveillance, ensuring rapid dog vaccination response to confirmed cases and generating actionable data for targeted interventions. This shift underscores the value of contextual tailoring in implementation research while maintaining the One Health ethos. The IBCM approach evaluated in this study differs from the Goa model, which primarily uses public and veterinary reporting through a hotline mechanism.^29^ Our model initiates IBCM within the health sector by identifying cases from patients presenting with dog-associated bite injuries, an approach that may allow a more comprehensive One Health collaboration. It also may capture more events given the likelihood of patient reporting through health facilities.

IBCM has been found to greatly increase case detection of rabies across a range of settings. In Tanzania, increased detection of probable rabid dogs was attributed to the collaboration fostered between health workers and veterinary officers.^5^ In Albay Province of the Philippines, implementation of IBCM quadrupled rabies case detection compared with passive surveillance,^30^ similar to the experiences from Uganda.^31^

In terms of improving best practices for PEP administration, IBCM has potential to reduce costs through judicious use of PEP and to improve adherence to PEP regimens among those exposed to rabies.^32^ Critically, through systematic sharing of information across sectors to ensure coordinated action, our implementation study aims to increase both cross-sectoral coordination and community engagement.

The stepped-wedge design is suited to this evaluation, allowing phased introduction across districts so that all eventually benefit while providing comparative data on effectiveness and feasibility. This design is ethically favourable and operationally pragmatic, capturing heterogeneity in local contexts and enabling iterative learning during rollout.^33–35^ Implementation research methods allow systematic exploration of facilitators, barriers and contextual factors influencing integration into existing health and veterinary systems, ensuring that findings are not only about effectiveness but also about scalability and sustainability.^36 37^

The protocol as designed has some limitations. The plan to enrol the first two eligible patients each day allows for detailed follow-up, due to operational and human resource constraints in busy primary care settings. This strategy shall ensure feasibility within routine clinic workflows, minimise disruption to service delivery and provides a standardised and consistent recruitment procedure across sites. While random selection of patients would be methodologically preferable, this was not considered operationally feasible in real-time clinical settings and may delay case capture and follow-up procedures. The chosen approach reduces investigator-driven convenience selection, but does not eliminate selection bias. Importantly, hospital-triggered and community-triggered reporting of rabies events ensure that surveillance and response to probable and confirmed cases are not restricted to enrolled participants.

The study is ambitious in scope and will require the concurrent implementation of multiple activities, including qualitative data collection, surveillance, field investigations, ring vaccination, implementation evaluation and economic assessment. The study feasibility will be strengthened by a collaborative One Health framework involving multiple partners, each contributing complementary expertise and responsibilities. Dedicated teams will be assigned to specific components of the study, thereby reducing the operational burden on individual investigators and enabling the parallel implementation of activities. Regular coordination meetings, standardised operating procedures, staff training and monitoring mechanisms will be instituted to ensure effective implementation, maintain data quality and support fidelity across study sites.

We anticipate challenges, including under-reporting of animal rabies cases, variable intersectoral coordination and logistical delays in laboratory confirmation. Community-level concerns, including trust in veterinary reporting and responsiveness of rapid dog vaccination teams, may also influence outcomes. These limitations, which are inherent to the implementation of real-world public health programmes, will be considered when interpreting the study findings and will be documented to inform adaptation and scaling.

## Ethics and dissemination

The study is approved by the WHO Ethics Committee (2024.9.Ind), Kerala University of Health Sciences (AX04/SOP07A/V2) and the ethics committee of Government Medical College, Thiruvananthapuram (HEC No.12/06/2024/MCT). Permissions have been obtained from the departments of health (GO(Rt)No. 2287/2024/H&FWD), animal husbandry (DAHO/TVM/409/2025-g) and local self-government (LSGD/PD/6303/2024-PH2). Approval was also obtained from the Government of India (F.No.P-29010/41/2024-DM) for international collaboration.

Informed written consent (online supplemental file 6) will be obtained from all participants and stakeholders. Only de-identified data, with no personal identifiers, will be used for analysis. Personal information will solely be used to facilitate appropriate follow-up and to ensure the provision of necessary care to patients, families, animals and communities involved. Animal welfare will be prioritised during the study.

The study protocol has been reviewed and aligned with the Standards for Reporting Implementation Studies (StaRI) guidelines to ensure comprehensive reporting of all relevant components (online supplemental file 7)

Dissemination will occur through policy briefs, stakeholder workshops and peer-reviewed publications, with special emphasis on engaging state-level One Health platforms to translate findings into policy and programmatic action. Amendments to the study protocol, including potential termination, will be reviewed and approved by the institutional ethics committee, with due regard for participant safety, transparency and reporting requirements.

## Supplementary material

10.1136/bmjopen-2026-117497 online supplemental file 1

10.1136/bmjopen-2026-117497 online supplemental file 2

10.1136/bmjopen-2026-117497 online supplemental file 3

10.1136/bmjopen-2026-117497 online supplemental file 4

10.1136/bmjopen-2026-117497 online supplemental file 5

10.1136/bmjopen-2026-117497 online supplemental file 6

10.1136/bmjopen-2026-117497 online supplemental file 7
