# Supplementary material for: Integrated bite case management within a One Health framework for rabies elimination at the primary care level in Kerala, India: an implementation research protocol using a stepped-wedge cluster randomised design
Source: BMJ Open. 2026 Sep 23;16(9):e117497. doi: 10.1136/bmjopen-2026-117497 (PMC13629920; doi:10.1136/bmjopen-2026-117497)
Supplement: Supplementary file 1 [file bmjopen-16-9-s001.pdf]

IBCM Prevelance VERSION 5

Welcome to the survey, this is a survey to collect information about the human anilmal exposures in our community. If you are willing to give the details kindly the sign the informed consent form and we shall proceed with the survey.

1. Willing to participate in the study?

- ○ Yes
- ○ No

1.1 If no, are you willing to provide the basic details

- ○ Yes
- ○ No

Thank you-

Basic information-

2. What is the name of the Block?

3. What is the name of the PHC?

4. study setting-

rural/tribal/urban/coastal

5. Location-

latitude (x.y °)

longitude (x.y °)

altitude (m)

accuracy (m)

5.1 What is the name of the Block?

5.2 What is the name of the PHC?

5.3 Study setting-

-

rural/tribal/urban/coastal

6. location-

latitude (x.y °)

longitude (x.y °)

altitude (m)

accuracy (m)

Household information-

7. Please enter unique household ID

First two letter of block, second two letter ward, third three letters house

8. Please enter data collectors id

Write your first name

9. Please enter ward number

10. Please enter house number-

11. Please enter number of members in a household

12. Please enter the phone number of 2 persons in a household
Upto 10 digits

---

13. Second phone number
Upto 10 digits

---

14. Date of data collection

---

15. Any pet animals owned by the household
yes/no. If yes, go to form B1

- ○ Yes
- ○ No

16. Any animal with community ownership in the neighbourhood
yes/no, if yes go to form B2

- ○ Yes
- ○ No

17. Death in the last 5 years

- ○ Yes
- ○ No
- ○ Don't know

17.1 Reason for death

- ○ Death due to encephalitis
- ○ Death due to rabies
- ○ Others specify

If other-

17.2 cause of encephalitis

- ○ Known
- ○ Unknown

if know-

Individual information-

Individual 1

*** 18. please enter unique individual id-**

*** 19. please enter the name of the individual-**

*** 20. please select gender**

male/female/others

- ○ male
- ○ female
- ○ others

*** 21. Age in years-**

*** 22. Occupation**

- ○ Student
- ○ Home maker
- ○ Unemployed
- ○ Unskilled worker
- ○ Semi skilled worker
- ○ Skilled worker
- ○ Clerical, shop owner, farm
- ○ Semi-professional
- ○ Professional

*** 23. education**

- ○ Illiterate
- ○ Primary school
- ○ Middle school
- ○ High school
- ○ Intermediate or diploma
- ○ Graduate/Post-graduate
- ○ Professional

24.Annual income-

as mentioned in the ration card/according to card colour

---

25. Other co-morbidities/illnesses

DM, HT, CAD,Others-please specify

- ☐ DM
- ☐ HT
- ☐ CAD

- ☐ Others
- ☐ No

26. If Others

---

27. Pre exposure vaccination

yes/no

- ○ Yes
- ○ No

28. history of animal bite

yes/no, if yes go to form D

- ○ Yes
- ○ No

Exposed Individual-

29. How many times have you been exposed to bite?-

---

30. When was the bite

Within one year, ------years

- ○ Within one year
- ○ 2-3 years back
- ○ 4-5years back
- ○ >5 years back

31. Nature of animal

Domestic/stray/wild

- ○ Domestic
- ○ Stray
- ○ Wild
- ○ undetermined

32. Type of animal

Dog/Cat/Others-specify

- ○ Dog
- ○ Cat
- ○ Others, please specify….

if others specify-

---

33. Symptoms in Animal

ALPHA

- ☐ Aggression
- ☐ Lethargy
- ☐ Paralysis
- ☐ Hypersalivation
- ☐ Abnormal vocalisation

☐ No symptoms

☐ Other symptoms, please specify…….

Other symptoms, please specify…….-

---

34. Did the animal have a contact with a rabies animal in the last 12 months

yes/no

- ○ Yes
- ○ No
- ○ Don't know

35. Is the animal alive

yes/no/don't know

- ○ Yes
- ○ No
- ○ Don't know

35.1 If not, killed or dead

- ○ Killed
- ○ Died

35.2. Died or killed after how many days of exposure

---

36. consulted veterinary doctor

yes/no/don't know

- ○ Yes
- ○ No
- ○ Don't know

37. Animal quarantined

yes/no/don't know

- ○ Yes
- ○ No
- ○ Don't know

39. Animal tested for rabies

yes/no/don't know

- ○ Yes
- ○ No
- ○ Don't know

40. If tested, what is the report of the test

rabid, not rabid, don't know

- ○ rabid
- ○ not rabid
- ○ Don't know

41. Bite

provoked/unprovoked

- ○ provoked

- ○ unprovoked

42. What is the nature of the exposure?

lick/bite/scratch

- ○ bite
- ○ lick
- ○ scratch

43. Please mention the site of exposure

head and neck/upper limb/lower limb/trunk/genitals

- ☐ head and neck
- ☐ upper limb
- ☐ lower limb
- ☐ trunk
- ☐ genitals

44. Please mention the wound category

I/II/III

- ○ I
- ○ II
- ○ III

45. Availability of documents

yes/no

- ○ Yes
- ○ No

46. Did you seek care?

yes/no

- ○ Yes
- ○ No

47. Kindly specify the reasons for not seeking care-

---

48. How many days did you take to seek treatment from the day of exposure?

---

49. Which sector did you consult?

Government/ Private

- ○ Government
- ○ Private

50. Which system of medicine did you seek care

Modern/others, if others, pls specify

- ○ Modern
- ○ Others, pls specify…….

others specify-

---

51. Did you take antirabies vaccine?

yes/no

- Yes
- No

52. What is the status of ARV?

completed/not completed

- completed
- not completed

53. Did you take rabies immunoglobulin

yes/no

- Yes
- No

54. What is the type of immunoglobulin taken?

Equine/human

- Equine
- Human
- MAB

55. Any allergy to test dose?

yes/no

- Yes
- No

56. Type of prophylaxis taken

pre/post/re

- Pre exposure
- Post exposure
- Re exposure

57. Had allergy to full dose?

- Yes
- No

58. How satisfied are you with the services provided to you on a scale of 1-10?-

Any number from1-10, with 1 being the lowest and 10,highest

---

59. How satisfied are you with the services provided to the animal that bit you on a scale of 1-10?-

Any number from1-10, with 1 being the lowest and 10,highest ; 0 is animal not taken for services

---

60. Kindly rate the animal welfare during that time on a scale of 1-10-

Any number from1-10, with 1 being the lowest and 10,highest ; not able to say or not assessed give "0"

Pet/domestic animal details-

61. Did you have pet/domestic animals

- ○ Yes
- ○ No

62. Select the pet/domestic animals

- ☐ Dog
- ☐ Cat
- ☐ Cattle
- ☐ Others

63. How many Dogs?

____

64. How many Cat?

____

65. How many Cattle?

____

66. Others how many ?

____

Dog-

67. Is this pet or community owned

- ○ Pet
- ○ Community owned

67. Age

---

68. Gender

- ○ Male
- ○ Female

69. . Vaccination status

- ○ Vaccinated completely and taking boosters
- ○ Vaccinated completely
- ○ Partially vaccinated
- ○ Unvaccinated

70. . Keep vaccination documentation (card/certificate)

- ○ Yes
- ○ No

71. Whether taken licensure/registration with LSGD/AHD

- ○ Yes
- ○ No

72. Whether spaying/neutering done

- ○ Yes
- ○ No
- ○ Don't know

73. Attacked humans in the past 12 months

- ○ Yes
- ○ No
- ○ Don't know

74. Has it been attacked by other animals in past 12 months

- ○ Yes
- ○ No
- ○ Don't know

75. Vaccination following attack (write number of vaccines taken)-

---

76. How many times has the animal been taken to a veterinary doctor in the last 12 months-

---

Cat-

77. Is this pet or community owned

- Pet
- Community owned

78. age-

---

79. Gender

- Male
- Female

80. Vaccination status

- Vaccinated completely and taking boosters
- Vaccinated completely
- Partially vaccinated
- Unvaccinated

81. Keep vaccination documentation (card/certificate)

- Yes
- No

82. Whether taken licensure/registration with LSGD/AHD

- Yes
- No

83. Whether spaying/neutering done

- Yes
- No
- Don't know

84. Attacked humans in the past 12 months

- Yes
- No
- Don't know

85. Has it been attacked by other animals in past 12 months

- Yes
- No
- Don't know

86. Vaccination following attack (write number of vaccines taken)-

---

87. How many times has the animal been taken to a veterinary doctor in the last 12 months-

---

cattle-

88. Age-

---

89. Gender

- ○ Male
- ○ Female

90. Vaccination status

- ○ Vaccinated completely and taking boosters
- ○ Vaccinated completely
- ○ Partially vaccinated
- ○ Unvaccinated

91. Keep vaccination documentation (card/certificate)

- ○ Yes
- ○ No

92. Whether taken licensure/registration with LSGD/AHD

- ○ Yes
- ○ No

93. Whether spaying/neutering done

- ○ Yes
- ○ No
- ○ Don't know

94. Attacked humans in the past 12 months

- ○ Yes
- ○ No
- ○ Don't know

95. Has it been attacked by other animals in past 12 months

- ○ Yes
- ○ No
- ○ Don't know

96. Vaccination following attack (write number of vaccines taken)-

---

97. How many times has the animal been taken to a veterinary doctor in the last 12 months-

---

others-

98. what type?-

---

99. Age-

---

100. Gender

- ○ Male
- ○ Female

101. Vaccination status

- ○ Vaccinated completely and taking boosters
- ○ Vaccinated completely
- ○ Partially vaccinated
- ○ Unvaccinated

102. Keep vaccination documentation (card/certificate)

- ○ Yes
- ○ No

103. Whether taken licensure/registration with LSGD/AHD

- ○ Yes
- ○ No

104. Whether spaying/neutering done

- ○ Yes
- ○ No
- ○ Don't know

105. Attacked humans in the past 12 months

- ○ Yes
- ○ No
- ○ Don't know

106. Has it been attacked by other animals in past 12 months

- ○ Yes
- ○ No
- ○ Don't know

107. Vaccination following attack (write number of vaccines taken)-

---

108. How many times has the animal been taken to a veterinary doctor in the last 12 months-

---

Remarks if any-
