# Supplementary material for: Integrated bite case management within a One Health framework for rabies elimination at the primary care level in Kerala, India: an implementation research protocol using a stepped-wedge cluster randomised design
Source: BMJ Open. 2026 Sep 23;16(9):e117497. doi: 10.1136/bmjopen-2026-117497 (PMC13629920; doi:10.1136/bmjopen-2026-117497)
Supplement: Supplementary file 2 [file bmjopen-16-9-s002.pdf]

IBCM Trigger 20250303 version05

Welcome to Trigger Event and Follow-up Data Capture Form

Trigger event

Consent provided

- ○ Yes
- ○ No

Event ID

First 4 letters of the centre followed by patient serial number

---

Cluster

Phase

phase 1, phase 2, cohort, dropout

- ○ Phase 1
- ○ Phase 2

Notified by

- ○ Community lay person
- ○ Health worker
- ○ Vet. Personnel
- ○ Owner of animal
- ○ Self-reported

Name of the linked PHC

Name of the linked Veterinary hospital/Dispensary

Status of the Human and follow-up details: Include wound assessment and risk of PEP assessment

---

Human and follow-up details

Consent provided

- ○ Yes
- ○ No

Name:

Age:

Gender

- ◯ Male
- ◯ Female
- ◯ Other

Address

Ward number

Bite location

Phone number

% - In case of Children write the phone number and name of the Legally Accepted Representative

Education

- ◯ Illiterate
- ◯ Primary school
- ◯ Middle school
- ◯ High school
- ◯ Intermediate or diploma
- ◯ Graduate/Post-graduate
- ◯ Professional

Occupation

- ◯ Student
- ◯ Home maker
- ◯ Unemployed
- ◯ Unskilled worker
- ◯ Semi skilled worker
- ◯ Skilled worker
- ◯ Clerical, shop owner, farm
- ◯ Semi-professional
- ◯ Professional

COLOUR OF RATION CARD

____

Previously vaccinated for Rabies

- ◯ Yes
- ◯ No

If yes, how many years back

____

Type of vaccine

- ◯ IDRV
- ◯ IMRV

Course

- ◯ Completed
- ◯ Not completed

Comorbidities

- ☐ Diabetes
- ☐ Hypertension
- ☐ Coronory artery diseases
- ☐ others
- ☐ No

Others specify

____

Any condions of immunodeficiency

- ◯ Yes
- ◯ No

Any immunosuppressive drugs being taken

- ◯ Yes
- ◯ No

Weight

Height

Status of Dog and follow-up details

When was the exposure
.... hours back

Animal exposed to

- ◯ Dog

Type of Animal

- ◯ Domestic
- ◯ Stray
- ◯ Wild
- ◯ Undetermined
- ◯ Community owned

Symptoms in dog

- ☐ Do not ask routinely
- ☐ Aggression
- ☐ Lethargy
- ☐ Paralysis
- ☐ Hypersalivation
- ☐ Abnormal Vocalisation
- ☐ No symptoms
- ☐ Not known
- ☐ Other symptoms

Others specify

Did the dog have contact with a rabies animal within the last 12 months

- ◯ Do not ask routinely
- ◯ Yes
- ◯ No
- ◯ Unknown

Dog is now alive

- ◯ Do not ask routinely
- ◯ Yes
- ◯ No
- ◯ Unknown

If not alive

- ◯ Killed
- ◯ Died

Died/Killed after ..... days of exposure

____

Dog vaccination status

- ◯ Do not ask routinely
- ◯ Vaccinated
- ◯ Unvaccinated
- ◯ Unknown

Shown to veterinary doctor

- ◯ Do not ask routinely
- ◯ Yes
- ◯ No
- ◯ Unknown

Whether laboratory test done for rabies

- ◯ Do not ask routinely
- ◯ Yes
- ◯ No
- ◯ Unknown

If yes

- ◯ Positive
- ◯ Negative

wound examination details

1

wound numbers

- ◯ Wound 1
- ◯ Wound 2
- ◯ Wound 3
- ◯ other

Site:

- ◯ Head and Neck
- ◯ Upper limb
- ◯ Lower limb
- ◯ Trunk
- ◯ Genital

Transdermal:

- ◯ Yes
- ◯ No

Oozing of blood

- ◯ Yes
- ◯ No

Image

Click here to upload file. (< 100MB)

Study teams Checklist for WHO PEP risk assessment as per IBCM

Did the exposure occur >10 days ago?

- ◯ Yes
- ◯ No
- ◯ Unknown

Is the animal still alive?

- ◯ Yes
- ◯ No
- ◯ Unknown

Is the victim <14 years?

- Yes
- No
- Unknown

Does the bite victim have multiple deep wounds?

- Yes
- No
- Unknown

Does the bite victim have bites to highly innervated areas

head and neck, palm, external genitalia

- Yes
- No
- Unknown

Has the animal been tested for rabies and found positive/result awaited?

- Yes
- No
- Unknown

If yes

- Positive
- Negative
- result pending/inconclusive

Has animal been exposed to rabies in past 12 months

- Yes
- No
- Unknown

Has the animal been tested for rabies and found negative?

- Yes
- No
- Unknown

Did the animal show any signs of disease consistent with rabies?

- Yes
- No
- Unknown

Did the animal die within 10 days of the exposure?

- ◯ Yes
- ◯ No
- ◯ Unknown

Did the animal bite 2 or more people or animals?

- ◯ Yes
- ◯ No
- ◯ Unknown

Is the animal available for assessment and quarantine?

- ◯ Yes
- ◯ No
- ◯ Unknown

Is the animal up-to-date on vaccination?

- ◯ Yes
- ◯ No
- ◯ Unknown

Was the animal provoked to bite /scratch?

- ◯ Do not ask routinely
- ◯ Provoked
- ◯ Unprovoked

Current treatment details

Treatment seeking

- ◯ by self
- ◯ community trigger event initiated
- ◯ contact tracing

Wound Category

- ◯ |
- ◯ | |
- ◯ | | |

Wound washed with soap and water for 15 minutes

- ◯ Yes
- ◯ No

Anti- rabies Vaccina on(ARV) started

- ◯ Yes
- ◯ No

Type of vaccination

- ◯ IDRV
- ◯ IMRV

Treatment given in

- ◯ Fully in the clinic
- ◯ Partially done and reffered
- ◯ Fully reffered

Rabies immunoglobulin taken

- ◯ Yes
- ◯ No

If yes , type of immunoglobin

- ◯ Equine
- ◯ Human
- ◯ MAB

If yes, Allergey to test dose?

- ◯ Yes
- ◯ No

If yes, Allergey to full dose?

- ◯ Yes
- ◯ No

TT given:

- ◯ Yes
- ◯ No

Antibiotic given:

- ◯ Yes
- ◯ No

Name of antibiotic

____
