# Supplementary material for: Integrated bite case management within a One Health framework for rabies elimination at the primary care level in Kerala, India: an implementation research protocol using a stepped-wedge cluster randomised design
Source: BMJ Open. 2026 Sep 23;16(9):e117497. doi: 10.1136/bmjopen-2026-117497 (PMC13629920; doi:10.1136/bmjopen-2026-117497)
Supplement: Supplementary file 3 [file bmjopen-16-9-s003.pdf]

ASHA worker followup V1

Event ID

First 4 letters of the centre followed by patient serial number

---

Phase

- ○ Phase 1
- ○ Phase 2

Name of the patient

Name of the linked PHC

Contact tracing

Contact tracing done?

- ○ Yes
- ○ No
- ○ Unknown

How many other humans has the dog attacked?

How many other such humans traced and assessed for need of PEP?

No of community awareness sessions done linked to the case

Dog followup

Dog followedup

- ○ Yes
- ○ No

Day

- ○ Day 0
- ○ Day ---- on development of symptoms
- ○ Day 10

Quarantine/Isolation

- ◯ Yes
- ◯ No
- ◯ Unknown

If no , reason

Asymptomatic

- ◯ Yes
- ◯ No

Aggression

- ◯ Yes
- ◯ No

Lethargy

- ◯ Yes
- ◯ No

Paralysis

- ◯ Yes
- ◯ No

Hypersalivation

- ◯ Yes
- ◯ No

Abnormal vocalisation

- ◯ Yes
- ◯ No

others specify

Status:

- ◯ Healthy
- ◯ Suspect
- ◯ Probable
- ◯ Confirmed

Vaccination status

- ○ Unknown
- ○ incomplete primary
- ○ Complete primary
- ○ Completed primary and booster
- ○ Not willing for vaccination

How many other animals has the dog attacked?

____

Euthanised

- ○ Yes
- ○ No

Sample taken

- ○ Yes
- ○ No

Result

____

Patient followup

Patient followedup

- ○ Yes
- ○ No

Bite

- ○ Provoked
- ○ Unprovoked

Vaccination completed?

- ○ Yes
- ○ No

Day

- [ ] 0
- [ ] 3
- [ ] 7
- [ ] 14
- [ ] 28
- [ ] 42

Dates

yyyy-mm-dd

Vaccine given

- ( ) Yes
- ( ) No

Reported by

- ( ) Self
- ( ) Called

Any problems

Blood samples taken

- ( ) Yes
- ( ) No

Sample ID*

event ID + two digits to indicate the day of collected on 28/42

Result – Titres

Cost

costs of any purchase made

---

specify what and cost- separate questions

Travel for person

Loss of wages for person

Travel of accompanying person if any Loss of

Loss of wages for the accompanying person if any

Cost incurred to the treatment expenses related to dog?
Syringe, medicines,Registraion etc

Cost incurred to the transportation expenses related to dog?

Cost incurred to the loss of wage for person related to dog?

Rating

How satisfied are you with the services provided to you on a scale of 1-10?
Any number from1-10, with 1 being the lowest and 10,highest

How satisfied are you with the services provided to the animal that bit you on a scale of 1-10?
Any number from1-10, with 1 being the lowest and 10,highest ; 0 is animal not taken for services

Kindly rate the animal welfare during that time on a scale of 1-10
Any number from1-10, with 1 being the lowest and 10,highest ; not able to say or not assessed give "0"
