# Supplementary material for: Integrated bite case management within a One Health framework for rabies elimination at the primary care level in Kerala, India: an implementation research protocol using a stepped-wedge cluster randomised design
Source: BMJ Open. 2026 Sep 23;16(9):e117497. doi: 10.1136/bmjopen-2026-117497 (PMC13629920; doi:10.1136/bmjopen-2026-117497)
Supplement: Supplementary file 4 [file bmjopen-16-9-s004.pdf]

IDI _Schedule _Providers _version 1.0

Instructions: Parts 1, 2, and 3 are to be taken before the implementation of the program and part 4 will be to the same stakeholders after the program has been implemented.

Serial No: First two letters for the block, next two letters for PHC, next two letters will denote the category and the last two digits will be the numbers

Sl .No.	*Category & Code*	*Numbers*
1.	*Political Leaders - PL*	*8*
2.	*Program Managers - PM*	*2*
3.	*Medical Officers (DHS)- MH*	*6*
4.	*Medical Officers (AHD)- MA*	*6*
5.	*Health Care Workers (DHS)- HH*<br>*IBCM supervisor HH*	*6*
6.	*Health Care Workers (AHD) – HA*<br>*IBCM supervisor HA*	*6*
7.	*ASHA – AS - IBCM investigator (best and least performing blocks)*	*12*
8.	*Total 46*	

Serial No:

Identification details

Name: Age:

Sex:

Education:

Occupation:

Income:

Place of residence: Native place:

Designation:

Phone number

Interview commencement time: Concluding time :

Interview recorded: Yes/ No Label file with Serial No:

PART I : Introductory statement

My name is ……………., I work as…………………………………………………………………………………………………(Designation). I have come as an investigator for the study entitled "**Implementation of Integrated Bite Case Management (IBCM) as part of a one health program for Rabies at Primary health care level in Kerala: Cost-effectiveness, challenges and solutions".** The objective of the study is to find out how best (in the most cost-effective way) a coordinated system of surveillance between the health and animal sectors can be implemented, to identify the challenges in this and bring out solutions to move forward. We will use this information only for the benefit of the society. I would also appreciate your consent to making an audio recording of the interview, to ensure that I do not lose any information.

I'm willing to give this interview and I give consent for recording the same.

Name: Signature with date:

**We have coined a word Harmful Human Exposures (HHE) Instead of dog bites/dog exposures*

PART 2: General Questions

2.1. What do you think about the problem of harmful human exposure (HHE) to dogs in your area?

2.2. What do you think about the problem of rabies in your area?

2.3. Currently, what are the existing measures for controlling this problem?

2.4. Can you explain how an HHE is currently managed?

2.5. Can you explain how exposure of dogs to rabies/ rabid animals is currently managed?

2.6. According to you, what are the challenges faced in implementing this program?

2.7. In what ways has the current program given due importance /consideration to animal health?

2.8. According to you what would be the challenges if such a component is also incorporated into the program?

PART 3: IBCM implementation

3.1. Have you heard about IBCM? Could you explain your understanding?

3.2. Have you heard about post-exposure prophylaxis in which the number of doses/duration can be shortened?

TALK About the program

3.3. Who do you think would be the important stakeholders in implementing such a program?

3.4. How best do you think such a program can be implemented?

3.5. Can you identify what would be the extra cost of such of program?

Probe: HR, infrastructure, Logistics, Communication channels

Will have a table here and try to get a cost range

PART 4: After the implementation interview (the same stakeholders will be Interviewed)

4.1. Overall, how effective do you think the program has been?

What do you think were the challenges in the Implementation of IBCM? Could you suggest better ways of Implementation/solutions to overcome the challenges?

Probe: HR, Implementation, Logistics, Communication

Probe:

**Area**	**Rating of Success (score 1 to 10)/Effectiveness**	**Challenges**	**Solutions**
Acceptability			
Adoption			
Appropriateness			
Feasibility:			

Fidelity			
Penetration:			
Sustainability:			
Scalability:			

4.2. What is your perception of the cost-effectiveness of the program?
