# Supplementary material for: Integrated bite case management within a One Health framework for rabies elimination at the primary care level in Kerala, India: an implementation research protocol using a stepped-wedge cluster randomised design
Source: BMJ Open. 2026 Sep 23;16(9):e117497. doi: 10.1136/bmjopen-2026-117497 (PMC13629920; doi:10.1136/bmjopen-2026-117497)
Supplement: Supplementary file 5 [file bmjopen-16-9-s005.pdf]

FGD guide _version 1.0

Themes

Before IBCM implementation

1. The problem of Harmful human exposure to dogs
2. The problem of Rabies
3. Current programs to address this issue
4. Challenges and lacunae in the current program
5. One Health
6. IBCM & shortened vaccination regimen -idea – throw up for discussion and get suggestions on how best to implement

After IBCM implementation

1. About IBCM and shortened vaccination regimen
2. IBCM implementation experience
3. Effectiveness of program – score 1 to 10 (nominal group technique)
4. IBCM Implementation Challenges and Solutions

   Probes: Acceptability, Adoption, Appropriateness, Feasibility, Fidelity, Penetration, Sustainability, Scalability

5. Perceptions on Cost-effectiveness
