# Supplementary material for: Integrated bite case management within a One Health framework for rabies elimination at the primary care level in Kerala, India: an implementation research protocol using a stepped-wedge cluster randomised design
Source: BMJ Open. 2026 Sep 23;16(9):e117497. doi: 10.1136/bmjopen-2026-117497 (PMC13629920; doi:10.1136/bmjopen-2026-117497)
Supplement: Supplementary file 6 [file bmjopen-16-9-s006.pdf]

Informed Consent for *prevalence assessment* .

The title of the research is *" Implementation of Integrated Bite Case Management (IBCM) as part of a one health program for Rabies at Primary health care level in Kerala: Cost-effectiveness, challenges and solutions"*

Name of Principal Investigator: Dr Zinia T. Nujum

Name of Organization: School of Public Health, Kerala University of Health Sciences

Name of Sponsor: World Health Organisation – Tropical Disease Research

Name of Proposal and version: IBCM_Kerala version 4.1.

This Informed Consent Form has two parts:

- **Information Sheet (to share information about the research with you)**
- **Certificate of Consent (for signatures if you agree to take part)**

You will be given a copy of the full Informed Consent Form

PART I: Information Sheet

Introduction & Purpose of Research

I am Dr. Zinia T.Nujum, working as an Associate Professor at, School of Public Health, Kerala University of Health Sciences. I am trying to collaborate with the Health Services Department and Animal Husbandry Department to do a research to closely monitor the health of both humans and animals involved in a dog attack/ exposure and make it better .

Rabies is a major public health problem in Kerala. The disease is caused by a virus transmitted from a bite from an infected animal. If a person is infected by rabies that person will die for sure within a short time. Hence the only way to prevent rabies is by giving a series of vaccinations to people who are exposed to the risk of rabies following an animal bite/ exposure. There is currently a program that provides this.

We are trying to add a component in the program to check and monitor the health status of the dogs when there is such an event. We are also trying to vaccinate the dogs which have not been vaccinated. If the dogs develop symptoms of rabies' they will and be tested for rabies. Thus we are keen on the animals health and welfare as well.

This part of the study is being done to assess the extent of animal bites and related health seeking in humans and animals

I am going to give you information and invite you to be part of this research. Before you decide, you can talk to anyone you feel comfortable with about the research.

There may be some words that you do not understand. Please ask me to stop as we go through the information and I will take time to explain. If you have questions later, you can ask them to me or the study staff.

Type of Research

The research will be done in three phases. This is phase zero - During this phase there will be only collection of some information regarding you, your household members and animals in the household. This will be taken only if you are chosen from the community in which the research in taking place and it will be informed to you.

Participant selection

We are inviting all those in this part of the ward to participate in this research and enroll them if they are willing.

Do you know why we are asking you to take part in this study? Do you know what the study is about?

If the answer to the questions is yes, proceed to next section.

Voluntary Participation

Your participation in this research is entirely voluntary. It is your choice whether to participate or not. Whether you choose to participate or not, all the services you receive at this clinic will continue and nothing will change. You may change your mind later and stop participating even if you agreed earlier.

Do you know that you do not have to take part in this research study, if you do not wish to?

Do you have any questions?

Duration

This part of the study is only for today. We will not approach you again

Do you have any other questions?

Side Effects

Since we are not giving you any additional medicines, we do not expect any side effects to happen to you, because you are participating in this research.

Risks

There will be no additional risk because you are taking part in this research.

Benefits

The information acquired through this study will help us understand ways of preventing and controlling rabies with reduced costs. It will also enable coordinated work between the health and animal husbandry departments which look after human and animal health to bring about better human and animal health. This will in turn help to prevent and control the problem of rabies in the community in a better way.

Reimbursements

You will not be given any other money or gifts to take part in this research

Can you tell me if you have understood correctly the benefits that you will have if you take part in the study?

Do you have any other questions?

Confidentiality

With this research, something out of the ordinary is being done in your community. It is possible that if others in the community are aware that you are participating, they may ask you questions. We will not be sharing the identity of those participating in the research.

The information that we collect from this research project will be kept confidential. Information about you that will be collected during the research will be put away and no one but the researchers will be able to see it. Any information about you will have a number on it instead of your name. Only the researchers will know what your number is and we will lock that information up with a lock and key. It will not be shared with or given to anyone except your clinician.

Do you have any questions about them?

Sharing the Results

The knowledge that we get from doing this research will be shared with you through community meetings before it is made widely available to the public. Confidential information will not be shared. There will be small meetings in the community and these will be announced. After these meetings, we will publish the results so that other interested people may learn from our research.

Right to Refuse or Withdraw

You do not have to take part in this research if you do not wish to do so and refusing to participate will not affect your treatment at this clinic in any way. You will still have all the benefits that you would otherwise have at this clinic. You may stop participating in the research at any time that you wish without losing any of your rights as a patient here. Your treatment at this hospital will not be affected in any way.

Who to Contact

For any question or additional information on the study, you may contact the following:

- ✓ : Dr Zinia T. Nujum,

  TC 96/2827House No: 818, Prasanth Nagar, Medical College P.O, Thiruvananthapuram 695011 Kerala

  Phone: +919037356908; E-mail – drzinia@gmail.com

This proposal has been reviewed and approved by Human Ethics committee/ Institutional Review Board (HEC/IRB) of Kerala University of Health Sciences, which is a committee whose task it is to make sure that research participants are protected from harm.

It has also been reviewed by the Ethics Review Committee of the World Health Organization (WHO), which is funding the study.

Do you know that you do not have to take part in this study if you do not wish to? You can say No if you wish to?

Do you know that you can ask me questions later, if you wish to?

Do you know that I have given the contact details of the person who can give you more information about the study?

You can ask me any more questions about any part of the research study, if you wish to. Do you have any questions?

PART II: Certificate of Consent

I have read the preceding information. I have had the opportunity to ask questions about it and any questions that I have asked have been answered to my satisfaction. I consent voluntarily to participate as a participant in this research, Phase zero /Phase 1 /Phase 2

Print Name of Participant__________________

Signature of Participant ___________________

Date ___________________________

Day/month/year

If illiterate

I have witnessed the accurate reading of the consent form to the potential participant, and the individual has had the opportunity to ask questions. I confirm that the individual has given consent freely.

Print name of witness_____________________ AND Thumb print of participant

Signature of witness ______________________

Date ________________________

Day/month/year

Statement by the researcher/person taking consent

I have accurately read out the information sheet to the potential participant, and to the best of my ability made sure that the participant understands that the following will be done:

Information related to the participants will be collected using a questionnaire by asking questions and from medical records

I confirm that the participant was allowed to ask questions about the study, and all the questions asked by the participant have been answered correctly and to the best of my ability. I confirm that the individual has not been coerced into giving consent, and the consent has been given freely and voluntarily.

A copy of this ICF has been provided to the participant.

Print Name of Researcher/person taking the consent________________________

Signature of Researcher /person taking the consent__________________________

Date ___________________________

Day/month/year

Informed Consent form for the person from the community /attending the health centre with a history of exposure to dog, and who we are invited to participate in research on the cost-effectiveness of integrated bite case management and attempt to shorten the duration and number of doses of vaccination given to prevent rabies.

The title of the research is ***" Implementation of Integrated Bite Case Management (IBCM) as part of a one health program for Rabies at Primary health care level in Kerala: Cost-effectiveness, challenges and solutions"***

Name of Principal Investigator: Dr Zinia T. Nujum

Name of Organization: School of Public Health, Kerala University of Health Sciences

Name of Sponsor: World Health Organisation – Tropical Disease Research

Name of Proposal and version: IBCM_Kerala version 4.1.

This Informed Consent Form has two parts:

- **Information Sheet (to share information about the research with you)**
- **Certificate of Consent (for signatures if you agree to take part)**

You will be given a copy of the full Informed Consent Form

PART I: Information Sheet

Introduction & Purpose of Research

I am Dr. Zinia T.Nujum, working as an Associate Professor at, School of Public Health, Kerala University of Health Sciences. I am trying to collaborate with the Health Services Department and Animal Husbandry Department to do a research to closely monitor the health of both humans and animals involved in a dog attack/ exposure and make it better .

Rabies is a major public health problem in Kerala. The disease is caused by a virus transmitted from a bite from an infected animal. If a person is infected by rabies that person will die for sure within a short time. Hence the only way to prevent rabies is by giving a series of vaccinations to people who are exposed to the risk of rabies following an animal bite/ exposure. There is currently a program that provides this.

We are trying to add a component in the program to check and monitor the health status of the dogs when there is such an event. We are also trying to vaccinate the dogs which have not been vaccinated. If the dogs develop symptoms of rabies' they will undergo mercy killing and be tested for rabies. Thus we are keen on the animals health and welfare as well.

I am going to give you information and invite you to be part of this research. Before you decide, you can talk to anyone you feel comfortable with about the research.

There may be some words that you do not understand. Please ask me to stop as we go through the information and I will take time to explain. If you have questions later, you can ask them to me or the study staff.

Type of Research Intervention

The research will be done in three phases. During the first phase there will be only collection of some information regarding you, your household members and animals in the household. This will be taken only if you are chosen from the community in which the research in taking place and it will be informed to you.

If you are part of the second phase of the research, you will only have to provide some information about yourself and the kind of exposure you had with the dog. If you agree to participate in phase 3 in which we are trying to improve the care of humans and dogs involved in the exposure, some questions will be asked to you. You will receive the routine care from hospital. In addition, irrespective of the category of exposure, the health of the animal involved will be monitored. If the animal belongs to you we will take a separate consent for that.

We will require information on the exposure to the dog and some information about the dog. The concerned dog will also be followed up and its health status monitored by a competent person. If the dog is not vaccinated, we will attempt to vaccinate the dog after the observation period. If the dog develops symptoms of rabies' they will undergo mercy killing and be tested for rabies.

Participant selection

We are inviting all those who are attending this clinic seeking care after exposure to dog to participate in this research. We will also mobilize people from the community who have been exposed to an attack by a dog to seek care and enroll them if they are willing.

Do you know why we are asking you to take part in this study? Do you know what the study is about?

If the answer to the questions is yes, proceed to next section.

Voluntary Participation

Your participation in this research is entirely voluntary. It is your choice whether to participate or not. Whether you choose to participate or not, all the services you receive at this clinic will continue and

nothing will change. You may change your mind later and stop participating even if you agreed earlier.

Do you know that you do not have to take part in this research study, if you do not wish to?

Do you have any questions?

Duration

The data collection for this research takes place over 24 months in total, but you will have to cooperate and be involved with us only for 28 days. During that time, it will be necessary for you to come to the clinic on specific dates. Each visit to this clinic will take about one hour. We will schedule these visits. In total, you will be asked to come 4 times to the clinic, which is also the number of times you would have to come fir vaccination, even if you are not part of the study.

Can you tell me if you remember the number of times that we are asking you to come to the hospital to complete the research project?

Do you have any other questions? Do you want me to go through the procedures again?

Side Effects

Since we are not giving you any additional medicines, we do not expect any side effects to happen to you, because you are participating in this research.

Risks

There will be no additional risk because you are taking part in this research.

Benefits

If you participate in this research, you will have the following benefits. You and the dog to which you are exposed will be taken care of well. Any interim problems or queries you have related to your exposure; you can contact us for guidance and help. We will help you in the best way we can, direct you to someone else, or clarify your queries to the best of our knowledge. The information acquired through this study will help us understand ways of preventing and controlling rabies with reduced costs. It will also enable coordinated work between the health and animal husbandry departments which look after human and animal health to bring about better human and animal health. This will in turn help to prevent and control the problem of rabies in the community in a better way.

Reimbursements

You will not be given any other money or gifts to take part in this research

Can you tell me if you have understood correctly the benefits that you will have if you take part in the study?

Do you have any other questions?

Confidentiality

With this research, something out of the ordinary is being done in your community. It is possible that if others in the community are aware that you are participating, they may ask you questions. We will not be sharing the identity of those participating in the research.

The information that we collect from this research project will be kept confidential. Information about you that will be collected during the research will be put away and no one but the researchers will be able to see it. Any information about you will have a number on it instead of your name. Only the researchers will know what your number is and we will lock that information up with a lock and key. It will not be shared with or given to anyone except your clinician.

Did you understand the procedures that we will be using to make sure that any information that we as researchers collect about you will remain confidential?

Do you have any questions about them?

Sharing the Results

The knowledge that we get from doing this research will be shared with you through community meetings before it is made widely available to the public. Confidential information will not be shared. There will be small meetings in the community and these will be announced. After these meetings, we will publish the results so that other interested people may learn from our research.

Right to Refuse or Withdraw

You do not have to take part in this research if you do not wish to do so and refusing to participate will not affect your treatment at this clinic in any way. You will still have all the benefits that you would otherwise have at this clinic. You may stop participating in the research at any time that you wish without losing any of your rights as a patient here. Your treatment at this hospital will not be affected in any way.

Who to Contact

For any question or additional information on the study, you may contact the following:

✓ : Dr Zinia T. Nujum,

TC 96/2827House No: 818, Prasanth Nagar, Medical College P.O, Thiruvananthapuram 695011

Kerala

Phone: +919037356908; E-mail – drzinia@gmail.com

This proposal has been reviewed and approved by Human Ethics committee/ Institutional Review Board (HEC/IRB) of Kerala University of Health Sciences, which is a committee whose task it is to make sure that research participants are protected from harm.

It has also been reviewed by the Ethics Review Committee of the World Health Organization (WHO), which is funding the study.

Do you know that you do not have to take part in this study if you do not wish to? You can say No if you wish to?

Do you know that you can ask me questions later, if you wish to?

Do you know that I have given the contact details of the person who can give you more information about the study?

You can ask me any more questions about any part of the research study, if you wish to. Do you have any questions?

PART II: Certificate of Consent

I have read the preceding information. I have had the opportunity to ask questions about it and any questions that I have asked have been answered to my satisfaction. I consent voluntarily to participate as a participant in this research, Phase 1/Phase 2 /Phase 3

Print Name of Participant__________________

Signature of Participant ___________________

Date ___________________________

Day/month/year

If illiterate

I have witnessed the accurate reading of the consent form to the potential participant, and the individual has had the opportunity to ask questions. I confirm that the individual has given consent freely.

Print name of witness_____________________ AND Thumb print of participant

Signature of witness ______________________

Date ________________________

Day/month/year

Statement by the researcher/person taking consent

I have accurately read out the information sheet to the potential participant, and to the best of my ability made sure that the participant understands that the following will be done:

Information related to the participants will be collected using a questionnaire by asking questions and from medical records

I confirm that the participant was allowed to ask questions about the study, and all the questions asked by the participant have been answered correctly and to the best of my ability. I confirm that the individual has not been coerced into giving consent, and the consent has been given freely and voluntarily.

A copy of this ICF has been provided to the participant.

Print Name of Researcher/person taking the consent________________________

Signature of Researcher /person taking the consent__________________________

Date ___________________________

Day/month/year

ഈ പഠനത്തിൻറെ പേര് : ഇമ്പ്ലിമെന്റേഷൻ ഓഫ് ഇൻഡഗ്രേറ്റഡ് ബൈറ്റ് കേസ് മാനേജ്മെൻറ് ആസ് പാർട്ട് ഓഫ് വൺ ഹെൽത്ത് പ്രോഗ്രാം ഫോർ റേബീസ് പ്രൈമറി ഹെൽത്ത് സെൻററർ ലെവൽ ഇൻ കേരള : കോസ്റ്റ് ഇഫക്ട് എഫക്ടിവെൻസ് ചാല്ലെങ്സ് ആൻഡ് സൊല്യൂഷൻ

പഠനത്തിൽ പങ്കെടുക്കുന്നവർക്കായുള്ള സമ്മതപത്രം

പ്രധാന ഗവേഷകയുടെ പേര് : *ഡോക്ടർ സിനിയ റ്റി. നുജും*

സ്ഥാപനത്തിൻറെ പേര്: *സ്കൂൾ ഓഫ് പബ്ലിക് ഹെൽത്ത് കേരള യൂണിവേഴ്സിറ്റി ഓഫ് ഹെൽത്ത് സയൻസ്*

ഈ പഠനം സ്പോൺസർ ചെയ്യുന്നത് :*ലോകാരോഗ്യ സംഘടനയുടെ ട്രോപ്പിക്കൽ ഡിസീസ് റിസർച്ച്*

ഈ സമ്മതപത്രത്തിന് രണ്ട് ഭാഗങ്ങളുണ്ട്

ഒന്ന്: ഈ പഠനത്തെ കുറിച്ചുള്ള വിവരം നൽകുന്ന വിവരണ രേഖ

രണ്ട്: സമ്മതം വാങ്ങുന്നതിനായുള്ള സമ്മതപത്രംnuju

നിങ്ങൾക്ക് ഇതിൻറെ ഒരു കോപ്പി നൽകുന്നതാണ്

ഭാഗം ഒന്ന് : വിവരണ രേഖ

ആമുഖം, ഉദ്ദേശം

എൻറെ പേര് ഡോ. സീനിയ ടി നുജും. ഞാൻ സ്കൂൾ ഓഫ് പബ്ലിക് ഹെൽത്തിൽ ജോലി ചെയ്യുകയാണ്.

റാബീസ് കേരളത്തിൽ ഒരു പ്രധാനപ്പെട്ട ആരോഗ്യ പ്രശ്നനമാണ്. റാബീസ് അണുബാധ ഉണ്ടായാൽ ആ വ്യക്തി തീർച്ചയായും മരണപ്പെടും. ഈ രോഗം വരാതിരിക്കാൻ പ്രതിരോധ മാർഗ്ഗങ്ങൾ മാത്രമേ നിലവിലുള്ളു. ഇതിനായി സർക്കാർ ഒരു പരിപാടി നടപ്പിലാക്കുന്നുണ്ട്.

കടിക്കുന്ന /ഉപദ്രവിക്കുന്ന നായ്ക്കളിൽ എല്ലാത്തിനും പേവിഷബാധ ഇല്ല കടിച്ച നായയുടെ ആരോഗ്യ സ്ഥിതി പരിശോധിച്ച് അതനുസരിച്ചു തീരുമാനം എടുക്കുകയാണെങ്കിൽ ചിലപ്പോഴെങ്കിലും കുത്തിവെപ്പിൻറെ എണ്ണം കുറക്കാൻ സാധിക്കും. അതേപോലെ പട്ടിയുടെ ആരോഗ്യവും കൂടി മനുഷ്യരുടെ കാര്യം നോക്കുന്നത് പോലെ നോക്കി അതിനു വാക്സിൻ കൊടുക്കേണ്ടതുണ്ട്. ഈ ഗവേഷണത്തിലൂടെ. അവർക്ക് രോഗലക്ഷണങ്ങൾ ഉണ്ടാവുകയാണെങ്കിൽ അവരെ ടെസ്റ്റ് ചെയ്ത് പേ വിഷബാധ ഉണ്ടോ എന്നും നോക്കുന്നതാണ്. മേല്പറഞ്ഞ എല്ലാ സംവിധാനവും നിലവിലെ പ്രധിരോധ പരിപാടിയോടുകൂടി ഒരുക്കുകയാണ് ഈ ഗവേഷണത്തിലൂടെ ചെയുന്നത്.

മൃഗങ്ങളുടെ കടിയുടെ വ്യാപ്തിയും മനുഷ്യരിലും മൃഗങ്ങളിലും ബന്ധപ്പെട്ട ആരോഗ്യം അന്വേഷിക്കുന്നതിനാണ് പഠനത്തിൻ്റെ ഈ ഭാഗം നടത്തുന്നത്

ഈ ഗവേഷണത്തിന്റെ കൂടുതൽ വിശദാംശങ്ങൾ നൽകി ഞാൻ നിങ്ങളെ ഈ പഠനത്തിലേക്ക് ക്ഷണിക്കുകയാണ്. എന്തെങ്കിലും മനസ്സിലാകാത്തത് ഉണ്ടെങ്കിൽ എപ്പോ വേണമെങ്കിലും എന്നോടോ എന്നോടൊപ്പം പ്രവർത്തിക്കുന്ന മറ്റു പ്രവർത്തകരോടോ ചോദിക്കാം.

ഗവേഷണത്തിന്റെ തരവും പങ്കാളിത്ത രീതിയും

മൂന്ന് ഘട്ടങ്ങളിലായാണ് ഗവേഷണം നടക്കുക. ഇത് ഘട്ടം പൂജ്യമാണ് - ഈ ഘട്ടത്തിൽ നിങ്ങളെയും നിങ്ങളുടെ കുടുംബാംഗങ്ങളെയും വീട്ടിലെ മൃഗങ്ങളെയും കുറിച്ചുള്ള ചില വിവരങ്ങളുടെ ശേഖരണം മാത്രമേ

ഉണ്ടാകൂ. ഗവേഷണം നടക്കുന്ന കമ്മ്യൂണിറ്റിയിൽ നിന്ന് നിങ്ങളെ തിരഞ്ഞെടുത്താൽ മാത്രമേ ഇത് എടുക്കൂ, അത് നിങ്ങളെ അറിയിക്കും.

പഠനത്തിൽ പങ്കെടുക്കാനുള്ള ക്ഷണം

നമ്മൾ നിങ്ങളെ പഠനത്തിലേക്ക് പങ്കാളിയാകാൻ ക്ഷണിക്കുകയാണ്.

എന്തുകൊണ്ടാണ് നിങ്ങളെ ഈ പഠനത്തിലേക്ക് ക്ഷണിക്കുന്നത് എന്ന് മനസ്സിലായോ? നിങ്ങൾക്ക് ഇതിനെക്കുറിച്ച് വേറെ എന്തെങ്കിലും കാര്യങ്ങൾ അറിയേണ്ടതുണ്ടോ? ഇല്ലെങ്കിൽ നമുക്ക് അടുത്ത കാര്യത്തിലേക്ക് കടക്കാം.

സ്വമേധയാ ഉള്ള പങ്കാളിത്തം

നിങ്ങളുടെ പങ്കാളിത്തം തികച്ചും സ്വതന്ത്രവും വ്യക്തിപരവുമാണ്. നിങ്ങൾ ഇനി പഠനത്തിൽ പങ്കെടുക്കാൻ ആഗ്രഹിക്കുന്നില്ല എങ്കിലും നിങ്ങൾക്ക് ലഭിക്കേണ്ട എല്ലാ സേവനങ്ങളും തുടർന്നും ലഭിക്കും. ഇടയിൽ നിങ്ങൾക്ക് എപ്പോഴെങ്കിലും ഗവേഷണത്തിലെ പങ്കാളിത്തം വേണ്ട എന്ന് തോന്നിയാൽ അപ്പോൾ നിങ്ങൾക്ക് ഈ പഠനത്തിൽ നിന്ന് പിൻവാങ്ങാം.

നിങ്ങൾ ആഗ്രഹിക്കുന്നില്ല എങ്കിൽ ഈ പഠനത്തിൽ പങ്കെടുക്കേണ്ട എന്നുള്ളത് നിങ്ങൾക്ക് മനസ്സിലായോ ?

ഈ ഘട്ടത്തിൽ നിങ്ങൾക്ക് എന്തെങ്കിലും ചോദ്യങ്ങളുണ്ടോ?

പാർശ്വ ഫലങ്ങൾ

നിങ്ങൾക്ക് ഈ ഗവേഷണത്തിന്റെ ഭാഗമായി അധിക മരുന്ന് നൽകുന്നില്ല എന്നുള്ളത് കൊണ്ട് പാർശ്വഫലങ്ങൾ പ്രതീക്ഷിക്കുന്നില്ല

ഈ ഗവേഷണത്തിൽ പങ്കെടുക്കുന്നത് കൊണ്ടുള്ള ഗുണങ്ങൾ

ഈ ഗവേഷണത്തിലൂടെ മനുഷ്യരുടെയും മൃഗങ്ങളുടെ ആരോഗ്യം ഒരുപോലെ പ്രാധാന്യം നൽകി ഇതിനുവേണ്ടി പ്രവർത്തിക്കുന്നവർ തമ്മിൽ ഏകോപിച്ച് പ്രവർത്തിക്കാൻ ഒരു സംവിധാനം ഉണ്ടാക്കാൻ സാധിക്കും. പ്രധിരോധ

പ്രവർത്തനങ്ങൾ കുറച്ചുകൂടി ഫലപ്രദമാകും എന്ന് ഞങ്ങൾ പ്രതീക്ഷിക്കുന്നു.

ഈ പഠനത്തിൽ പങ്കെടുക്കുന്നതിനായി നിങ്ങൾക്ക് പ്രത്യേക പൈസയോ പാരിതോഷികങ്ങളോ നൽകുന്നില്ല

ഗവേഷണത്തിൽ പങ്കെടുക്കുന്നത് കൊണ്ട് ഗുണങ്ങളെക്കുറിച്ച് നിങ്ങൾക്ക് മനസ്സിലായോ?

എന്തെങ്കിലും ചോദ്യങ്ങൾ ചോദിക്കാനുണ്ടോ?

നിങ്ങൾ തരുന്ന വിവരങ്ങൾ എല്ലാം രഹസ്യമായി സൂക്ഷി ക്കും. വ്യക്തിഗത വിവരങ്ങൾ ഒന്നും തന്നെ പുറത്ത് വിടുന്നതല്ല. വ്യക്തി ഗത വിവരം മാറ്റിവെച്ച ശേഷം മാത്രമേ വിലയിരുത്തൽ നടത്തുകയുള്ളു.

ഇത്രയും പറഞ്ഞതും മനസ്സിലായോ ചോദിക്കാൻ ഉണ്ടോ?

ഈ ഗവേഷണത്തിൽ കൂടി നമുക്ക് ലഭിക്കുന്ന വിവരം സമൂഹവുമായി പങ്കുവെയ്ക്കും. അത്തരത്തിൽ പങ്കുവയ്ക്കുമ്പോൾ ഒരിക്കലും വ്യക്തിഗത വിവരങ്ങൾ നൽകുന്നതല്ല

ഗവേഷണത്തിൽ പങ്കെടുക്കാൻ ആഗ്രഹിക്കുന്നില്ലെങ്കിൽ നിങ്ങൾക്ക് പങ്കെടുക്കേണ്ടതില്ല. നിങ്ങൾക്ക് ലഭിക്കേണ്ട എല്ലാ സേവനങ്ങളും തുടർന്നും ലഭിക്കും. എപ്പോൾ വേണമെങ്കിലും നിങ്ങള്ക്ക് നിങ്ങളുടെ പങ്കാളിത്തം പിൻവലിക്കാം. അത് നിങ്ങളുടെ ചികിത്സയെ ബാധിക്കില്ല

ഇതുമായി ബന്ധപ്പെട്ട് എന്തെങ്കിലും സംശയമുണ്ടെങ്കിൽ താഴെ പറയുന്ന വ്യക്തികളെ സമീപിക്കാവുന്നതാണ്.

ഡോ സീനിയ ടി നുജും,

TC 96/2827വീട് നമ്പർ: 818, പ്രശാന്ത് നഗർ, മെഡിക്കൽ കോളേജ് P.O,

തിരുവനന്തപുരം 695011 കേരളം

ഫോൺ: +919037356908; ഇ-മെയിൽ - drzinia@gmail.com

ഈ ഗവേഷണ രേഖ കേരള യൂണിവേഴ്സിറ്റി ഓഫ് ഹെൽത്ത് സയൻസ് എത്തിക്കൽ കമ്മിറ്റിയുടെ അനുവാദം ലഭിച്ചതാണ്.

ഇത് കൂടാതെ ലോകാരോഗ്യ സംഘടനയുടെ അനുവാദം ഈ പഠനത്തിന് ലഭിച്ചിട്ടുള്ളതാണ്. ലോകാരോഗ്യ സംഘടനയാണ് ഈ ഗവേഷണത്തിനുള്ള പണം നൽകിയിട്ടുള്ളത്.

ഈ ഘട്ടത്തിൽ എന്തെങ്കിലും ചോദ്യമുണ്ടെങ്കിൽ നിങ്ങൾക്ക് ചോദിക്കാം

ഈ പഠനത്തിൻറെ പേര് : ഇമ്പ്ലിമെന്റേഷൻ ഓഫ് ഇൻഡഗ്രേറ്റഡ് ബൈറ്റ് കേസ് മാനേജ്മെൻറ് ആസ് പാർട്ട് ഓഫ് വൺ ഹെൽത്ത് പ്രോഗ്രാം ഫോർ റേബീസ് പ്രൈമറി ഹെൽത്ത് സെൻറർ ലെവൽ ഇൻ കേരള : കോസ്റ്റ് ഇഫക്ട് എഫക്ടിവെൻസ് ചാല്ലെങ്സ് ആൻഡ് സൊല്യൂഷൻ

ഭാഗം രണ്ടു : സമ്മതപത്രം

ID:

ഞാൻ മേൽപ്പറഞ്ഞ വിവരം എല്ലാം വായിച്ചു മനസ്സിലാക്കി. ചോദ്യം ചോദിക്കാൻ ഉള്ള അവസരം എനിക്ക് കിട്ടിയിട്ടുണ്ട് അതിനുള്ള ഉത്തരം തൃപ്തി കരമായി കിട്ടിയിട്ടുണ്ട്.

ഗവേഷണത്തിൽ പങ്കെടുക്കാൻ സ്വമേധയാ സമ്മതിക്കുന്നു.

ഘട്ടം 0/ഘട്ടം 1 /ഘട്ടം 2

പങ്കെടുക്കുന്ന വ്യക്തിയുടെ പേര്:

പങ്കെടുക്കുന്ന വ്യക്തിയുടെ ഒപ്പ് തീയതി:

ഭാഗം രണ്ടു : സമ്മതപത്രം

ID:

ഞാൻ മേൽപ്പറഞ്ഞ വിവരം എല്ലാം വായിച്ചു മനസ്സിലാക്കി. ചോദ്യം ചോദിക്കാൻ ഉള്ള അവസരം എനിക്ക് കിട്ടിയിട്ടുണ്ട് അതിനുള്ള ഉത്തരം തൃപ്തി കരമായി കിട്ടിയിട്ടുണ്ട്.

ഗവേഷണത്തിൽ പങ്കെടുക്കാൻ സ്വമേധയാ സമ്മതിക്കുന്നു.

ഘട്ടം 0/ഘട്ടം 1 /ഘട്ടം 2

പങ്കെടുക്കുന്ന വ്യക്തിയുടെ പേര്:

പങ്കെടുക്കുന്ന വ്യക്തിയുടെ ഒപ്പ് തീയതി:

എഴുതാനും വായിക്കാനും അറിയാത്ത വ്യക്തിയാണെങ്കിൽ ഒരു സാക്ഷി

ഈ പഠനത്തെ കുറിച്ചുള്ള വിവരങ്ങൾ മനസ്സിലാക്കി, ഈ പഠനത്തിൽ പങ്കെടുക്കാൻ ഈ വ്യക്തി സ്വമേധയാ സമ്മതിച്ചിട്ടുള്ളതാണ്

സാക്ഷിയുടെ പേര്:

സാക്ഷിയുടെ ഉപ്പ്, തീയതി :

ഇവിടെ കൈവിരൽ അടയാളം:

പങ്കെടുക്കുന്ന വ്യക്തിയുടെ പേര്:

ID:

എഴുതാനും വായിക്കാനും അറിയാത്ത വ്യക്തിയാണെങ്കിൽ ഒരു സാക്ഷി

ഈ പഠനത്തെ കുറിച്ചുള്ള വിവരങ്ങൾ മനസ്സിലാക്കി, ഈ പഠനത്തിൽ പങ്കെടുക്കാൻ ഈ വ്യക്തി സ്വമേധയാ സമ്മതിച്ചിട്ടുള്ളതാണ്

സാക്ഷിയുടെ പേര്:

സാക്ഷിയുടെ ഉപ്പ്, തീയതി :

ഇവിടെ കൈവിരൽ അടയാളം:

പങ്കെടുക്കുന്ന വ്യക്തിയുടെ പേര്:

ID:

സമ്മതം വാങ്ങുന്ന ഗവേഷകൻ്റെ/വ്യക്തിയുടെ പ്രസ്താവന

പങ്കെടുക്കാൻ സാധ്യതയുള്ള വ്യക്തിക്ക് ഞാൻ വിവര ഷീറ്റ് കൃത്യമായി വായിച്ചു, കൂടാതെ ഇനിപ്പറയുന്നവ ചെയ്യുമെന്ന് പങ്കെടുക്കുന്നയാൾ മനസ്സിലാക്കുന്നുവെന്ന് ഉറപ്പാക്കുകയും ചെയ്തു:

1. പങ്കെടുക്കുന്നവരുമായി ബന്ധപ്പെട്ട വിവരങ്ങൾ ഒരു ചോദ്യാവലി ഉപയോഗിച്ച് ചോദ്യങ്ങൾ ചോദിച്ച് മെഡിക്കൽ രേഖകളിൽ നിന്ന് ശേഖരിക്കും

പഠനത്തെ കുറിച്ച് ചോദ്യങ്ങൾ ചോദിക്കാൻ പങ്കാളിയെ അനുവദിച്ചിട്ടുണ്ടെന്ന് ഞാൻ സ്ഥിരീകരിക്കുന്നു, പങ്കെടുക്കുന്നയാൾ ചോദിച്ച എല്ലാ ചോദ്യങ്ങൾക്കും കൃത്യമായും എൻ്റെ കഴിവിൻ്റെ പരമാവധിയിലും ഉത്തരം നൽകിയിട്ടുണ്ട്. സമ്മതം നൽകാൻ വ്യക്തിയെ നിർബന്ധിച്ചിട്ടില്ലെന്നും സമ്മതം സ്വതന്ത്രമായും സ്വമേധയാ നൽകിയിട്ടുണ്ടെന്നും ഞാൻ സ്ഥിരീകരിക്കുന്നു.

ഈ ഐസിഎഫിൻ്റെ ഒരു പകർപ്പ് പങ്കെടുക്കുന്നയാൾക്ക് നൽകിയിട്ടുണ്ട്.

സമ്മതം എടുക്കുന്ന ഗവേഷകൻ്റെ/വ്യക്തിയുടെ പേര്
അച്ചടിക്കുക______________________

ഗവേഷകൻ്റെ / സമ്മതം വാങ്ങുന്ന വ്യക്തിയുടെ ഒപ്പ്_________________________

തീയതി _________________________

ദിവസം/മാസം/വർഷം

ഈ പഠനത്തിൻറെ പേര് : ഇമ്പ്ലിമെന്റേഷൻ ഓഫ് ഇൻഡഗ്രേറ്റഡ് ബൈറ്റ് കേസ് മാനേജ്മെൻറ് ആസ് പാർട്ട് ഓഫ് വൺ ഹെൽത്ത് പ്രോഗ്രാം ഫോർ റേബീസ് പ്രൈമറി ഹെൽത്ത് സെൻറർ ലെവൽ ഇൻ കേരള : കോസ്റ്റ് ഇഫക്ട് എഫക്ടിവെൻസ് ചാല്ലെങ്സ് ആൻഡ് സൊല്യൂഷൻ

പഠനത്തിൽ പങ്കെടുക്കുന്നവർക്കായുള്ള സമ്മതപത്രം

പ്രധാന ഗവേഷകയുടെ പേര് : *ഡോക്ടർ സിനിയ റ്റി. നുജും*

സ്ഥാപനത്തിൻറെ പേര്: *സ്കൂൾ ഓഫ് പബ്ലിക് ഹെൽത്ത് കേരള യൂണിവേഴ്സിറ്റി ഓഫ് ഹെൽത്ത് സയൻസ്*

ഈ പഠനം സ്പോൺസർ ചെയ്യുന്നത് :*ലോകാരോഗ്യ സംഘടനയുടെ ട്രോപ്പിക്കൽ ഡിസീസ് റിസർച്ച്*

ഈ സമ്മതപത്രത്തിന് രണ്ട് ഭാഗങ്ങളുണ്ട്

ഒന്ന്: ഈ പഠനത്തെ കുറിച്ചുള്ള വിവരം നൽകുന്ന വിവരണ രേഖ

രണ്ട്: സമ്മതം വാങ്ങുന്നതിനായുള്ള സമ്മതപത്രംnuju

നിങ്ങൾക്ക് ഇതിൻറെ ഒരു കോപ്പി നൽകുന്നതാണ്

ഭാഗം ഒന്ന് : വിവരണ രേഖ

ആമുഖം, ഉദ്ദേശം

എൻറെ പേര് ഡോ. സീനിയ ടി നുജും. ഞാൻ സ്കൂൾ ഓഫ് പബ്ലിക് ഹെൽത്തിൽ ജോലി ചെയ്യുകയാണ്.

റാബീസ് കേരളത്തിൽ ഒരു പ്രധാനപ്പെട്ട ആരോഗ്യ പ്രശ്നനമാണ്. റാബീസ് അണുബാധ ഉണ്ടായാൽ ആ വ്യക്തി തീർച്ചയായും മരണപ്പെടും. ഈ രോഗം വരാതിരിക്കാൻ പ്രതിരോധ മാർഗ്ഗങ്ങൾ മാത്രമേ നിലവിലുള്ളു. ഇതിനായി സർക്കാർ ഒരു പരിപാടി നടപ്പിലാക്കുന്നുണ്ട്.

കടിക്കുന്ന /ഉപദ്രവിക്കുന്ന നായ്ക്കളിൽ എല്ലാത്തിനും പേവിഷബാധ ഇല്ല കടിച്ച നായയുടെ ആരോഗ്യ സ്ഥിതി പരിശോധിച്ച് അതനുസരിച്ചു തീരുമാനം എടുക്കുകയാണെങ്കിൽ ചിലപ്പോഴെങ്കിലും കുത്തിവെപ്പിൻറെ എണ്ണം കുറക്കാൻ സാധിക്കും. അതേപോലെ പട്ടിയുടെ ആരോഗ്യവും കൂടി മനുഷ്യരുടെ കാര്യം നോക്കുന്നത് പോലെ നോക്കി അതിനു വാക്സിൻ കൊടുക്കേണ്ടതുണ്ട്. ഈ ഗവേഷണത്തിലൂടെ. അവർക്ക് രോഗലക്ഷണങ്ങൾ ഉണ്ടാവുകയാണെങ്കിൽ അവരെ ടെസ്റ്റ് ചെയ്ത് പേ വിഷബാധ ഉണ്ടോ എന്നും നോക്കുന്നതാണ്. മേല്പറഞ്ഞ എല്ലാ സംവിധാനവും നിലവിലെ പ്രധിരോധ പരിപാടിയോടുകൂടി ഒരുക്കുകയാണ് ഈ ഗവേഷണത്തിലൂടെ ചെയുന്നത്.

മൃഗങ്ങളുടെ കടിയുടെ വ്യാപ്തിയും മനുഷ്യരിലും മൃഗങ്ങളിലും ബന്ധപ്പെട്ട ആരോഗ്യം അന്വേഷിക്കുന്നതിനാണ് പഠനത്തിൻ്റെ ഈ ഭാഗം നടത്തുന്നത്

ഈ ഗവേഷണത്തിന്റെ കൂടുതൽ വിശദാംശങ്ങൾ നൽകി ഞാൻ നിങ്ങളെ ഈ പഠനത്തിലേക്ക് ക്ഷണിക്കുകയാണ്. എന്തെങ്കിലും മനസ്സിലാകാത്തത് ഉണ്ടെങ്കിൽ എപ്പോ വേണമെങ്കിലും എന്നോടോ എന്നോടൊപ്പം പ്രവർത്തിക്കുന്ന മറ്റു പ്രവർത്തകരോടോ ചോദിക്കാം.

ഗവേഷണത്തിന്റെ തരവും പങ്കാളിത്ത രീതിയും

മൂന്ന് ഘട്ടങ്ങളിലായാണ് ഗവേഷണം നടക്കുക. പൂജ്യം ഘട്ടത്തിൽനിങ്ങളെയും നിങ്ങളുടെ കുടുംബാംഗങ്ങളെയും വീട്ടിലെ മൃഗങ്ങളെയും കുറിച്ചുള്ള ചില വിവരങ്ങളുടെ ശേഖരണം മാത്രമേ ഉണ്ടാകൂ. ഗവേഷണം നടക്കുന്ന കമ്മ്യൂണിറ്റിയിൽ നിന്ന് നിങ്ങളെ തിരഞ്ഞെടുത്താൽ മാത്രമേ ഇത് എടുക്കൂ, അത് നിങ്ങളെ അറിയിക്കും.

ഗവേശനത്തിൻ്റെ ഒന്നാം ഘട്ടത്തിൽ ആണു നിങ്ങൾ പങ്കെടുക്കുന്നതെങ്കിൽ നിങ്ങൾക്കുണ്ടായ പട്ടി കടിയുമയി സംബന്ധിച്ച വിവരണങ്ങൾ മാത്രം ചോദിക്കുകയുള്ളു . രണ്ടമത്തെ ഘട്ടത്തിൽ ആണു നിങ്ങൾ പങ്കെടുക്കുന്നതെങ്കിൽ ഈ വിവരണങ്ങൾ കൂടാതെ നിങ്ങളുടേയും നിങ്ങളെ ഉപദ്രവിച്ച നായയുടെ ആരോഗ്യം നിരീക്ഷിക്കും, ആവശ്യമേങ്കിൽ നായേ പരിശോധനെക്കു വിധേയമാക്കുകയും ചെയ്യും.

പഠനത്തിൽ പങ്കെടുക്കാനുള്ള ക്ഷണം

നമ്മൾ നിങ്ങളെ പഠനത്തിലേക്ക് പങ്കാളിയാകാൻ ക്ഷണിക്കുകയാണ്.

എന്തുകൊണ്ടാണ് നിങ്ങളെ ഈ പഠനത്തിലേക്ക് ക്ഷണിക്കുന്നത് എന്ന് മനസ്സിലായോ? നിങ്ങൾക്ക് ഇതിനെക്കുറിച്ച് വേറെ എന്തെങ്കിലും കാര്യങ്ങൾ അറിയേണ്ടതുണ്ടോ? ഇല്ലെങ്കിൽ നമുക്ക് അടുത്ത കാര്യത്തിലേക്ക് കടക്കാം.

സ്വമേധയാ ഉള്ള പങ്കാളിത്തം

നിങ്ങളുടെ പങ്കാളിത്തം തികച്ചും സ്വതന്ത്രവും വ്യക്തിപരവുമാണ്. നിങ്ങൾ ഇനി പഠനത്തിൽ പങ്കെടുക്കാൻ ആഗ്രഹിക്കുന്നില്ല എങ്കിലും നിങ്ങൾക്ക് ലഭിക്കേണ്ട എല്ലാ സേവനങ്ങളും തുടർന്നും ലഭിക്കും. ഇടയിൽ നിങ്ങൾക്ക് എപ്പോഴെങ്കിലും ഗവേഷണത്തിലെ പങ്കാളിത്തം വേണ്ട എന്ന് തോന്നിയാൽ അപ്പോൾ നിങ്ങൾക്ക് ഈ പഠനത്തിൽ നിന്ന് പിൻവാങ്ങാം.

നിങ്ങൾ ആഗ്രഹിക്കുന്നില്ല എങ്കിൽ ഈ പഠനത്തിൽ പങ്കെടുക്കേണ്ട എന്നുള്ളത് നിങ്ങൾക്ക് മനസ്സിലായോ ?

ഈ ഘട്ടത്തിൽ നിങ്ങൾക്ക് എന്തെങ്കിലും ചോദ്യങ്ങളുണ്ടോ?

പാർശ്വ ഫലങ്ങൾ

നിങ്ങൾക്ക് ഈ ഗവേഷണത്തിന്റെ ഭാഗമായി അധിക മരുന്ന് നൽകുന്നില്ല എന്നുള്ളത് കൊണ്ട് പാർശ്വഫലങ്ങൾ പ്രതീക്ഷിക്കുന്നില്ല

ഈ ഗവേഷണത്തിൽ പങ്കെടുക്കുന്നത് കൊണ്ടുള്ള ഗുണങ്ങൾ

ഈ ഗവേഷണത്തിലൂടെ മനുഷ്യരുടെയും മൃഗങ്ങളുടെ ആരോഗ്യം ഒരുപോലെ പ്രാധാന്യം നൽകി ഇതിനുവേണ്ടി പ്രവർത്തിക്കുന്നവർ തമ്മിൽ ഏകോപി ച്ച് പ്രവർത്തിക്കാൻ ഒരു സംവിധാനം ഉണ്ടാക്കാൻ സാധിക്കും. പ്രധിരോധ പ്രവർത്തനങ്ങൾ കുറച്ചുകൂടി ഫലപ്രദമാകും എന്ന് ഞങ്ങൾ പ്രതീക്ഷിക്കുന്നു.

ഈ പഠനത്തിൽ പങ്കെടുക്കുന്നതിനായി നിങ്ങൾക്ക് പ്രത്യേക പൈസയോ പാരിതോഷികങ്ങളോ നൽകുന്നില്ല

ഗവേഷണത്തിൽ പങ്കെടുക്കുന്നത് കൊണ്ട് ഗുണങ്ങളെക്കുറിച്ച് നിങ്ങൾക്ക് മനസ്സിലായോ?

എന്തെങ്കിലും ചോദ്യങ്ങൾ ചോദിക്കാനുണ്ടോ?

നിങ്ങൾ തരുന്ന വിവരങ്ങൾ എല്ലാം രഹസ്യമായി സൂക്ഷി ക്കും. വ്യക്തിഗത വിവരങ്ങൾ ഒന്നും തന്നെ പുറത്ത് വിടുന്നതല്ല. വ്യക്തി ഗത വിവരം മാറ്റിവെച്ച ശേഷം മാത്രമേ വിലയിരുത്തൽ നടത്തുകയുള്ളു.

ഇത്രയും പറഞ്ഞതും മനസ്സിലായോ ചോദിക്കാൻ ഉണ്ടോ?

ഈ ഗവേഷണത്തിൽ കൂടി നമുക്ക് ലഭിക്കുന്ന വിവരം സമൂഹവുമായി പങ്കുവെയ്ക്കും. അത്തരത്തിൽ പങ്കുവയ്ക്കുമ്പോൾ ഒരിക്കലും വ്യക്തിഗത വിവരങ്ങൾ നൽകുന്നതല്ല

ഗവേഷണത്തിൽ പങ്കെടുക്കാൻ ആഗ്രഹിക്കുന്നില്ലെങ്കിൽ നിങ്ങൾക്ക് പങ്കെടുക്കേണ്ടതില്ല. നിങ്ങൾക്ക് ലഭിക്കേണ്ട എല്ലാ സേവനങ്ങളും തുടർന്നും ലഭിക്കും. എപ്പോൾ വേണമെങ്കിലും നിങ്ങള്ക്ക് നിങ്ങളുടെ പങ്കാളിത്തം പിൻവലിക്കാം. അത് നിങ്ങളുടെ ചികിത്സയെ ബാധിക്കില്ല

ഇതുമായി ബന്ധപ്പെട്ട് എന്തെങ്കിലും സംശയമുണ്ടെങ്കിൽ താഴെ പറയുന്ന വ്യക്തികളെ സമീപിക്കാവുന്നതാണ്.

ഡോ സീനിയ ടി നുജും,

TC 96/2827വീട് നമ്പർ: 818, പ്രശാന്ത് നഗർ, മെഡിക്കൽ കോളേജ് P.O,
തിരുവനന്തപുരം 695011 കേരളം

ഫോൺ: +919037356908; ഇ-മെയിൽ - drzinia@gmail.com

ഈ ഗവേഷണ രേഖ കേരള യൂണിവേഴ്സിറ്റി ഓഫ് ഹെൽത്ത് സയൻസ് എത്തിക്കൽ കമ്മിറ്റിയുടെ അനുവാദം ലഭിച്ചതാണ്.

ഇത് കൂടാതെ ലോകാരോഗ്യ സംഘടനയുടെ അനുവാദം ഈ പഠനത്തിന് ലഭിച്ചിട്ടുള്ളതാണ്. ലോകാരോഗ്യ സംഘടനയാണ് ഈ ഗവേഷണത്തിനുള്ള പണം നൽകിയിട്ടുള്ളത്.

ഈ ഘട്ടത്തിൽ എന്തെങ്കിലും ചോദ്യമുണ്ടെങ്കിൽ നിങ്ങൾക്ക് ചോദിക്കാം

ഈ പഠനത്തിൻറെ പേര് : ഇമ്പ്ലിമെന്റേഷൻ ഓഫ് ഇൻഡഗ്രേറ്റഡ് ബൈറ്റ് കേസ് മാനേജ്മെൻറ് ആസ് പാർട്ട് ഓഫ് വൺ ഹെൽത്ത് പ്രോഗ്രാം ഫോർ റേബീസ് പ്രൈമറി ഹെൽത്ത് സെൻറർ ലെവൽ ഇൻ കേരള : കോസ്റ്റ് ഇഫക്ട് എഫക്ടിവെൻസ് ചാല്ലെങ്സ് ആൻഡ് സൊല്യൂഷൻ

ഭാഗം രണ്ടു : സമ്മതപത്രം

ID:

ഞാൻ മേൽപ്പറഞ്ഞ വിവരം എല്ലാം വായിച്ചു മനസ്സിലാക്കി. ചോദ്യം ചോദിക്കാൻ ഉള്ള അവസരം എനിക്ക് കിട്ടിയിട്ടുണ്ട് അതിനുള്ള ഉത്തരം തൃപ്തി കരമായി കിട്ടിയിട്ടുണ്ട്.

ഗവേഷണത്തിൽ പങ്കെടുക്കാൻ സ്വമേധയാ സമ്മതിക്കുന്നു.

ഘട്ടം 0/ഘട്ടം 1 /ഘട്ടം 2

പങ്കെടുക്കുന്ന വ്യക്തിയുടെ പേര്:

പങ്കെടുക്കുന്ന വ്യക്തിയുടെ ഒപ്പ് തീയതി:

ഭാഗം രണ്ടു : സമ്മതപത്രം

ID:

ഞാൻ മേൽപ്പറഞ്ഞ വിവരം എല്ലാം വായിച്ചു മനസ്സിലാക്കി. ചോദ്യം ചോദിക്കാൻ ഉള്ള അവസരം എനിക്ക് കിട്ടിയിട്ടുണ്ട് അതിനുള്ള ഉത്തരം തൃപ്തി കരമായി കിട്ടിയിട്ടുണ്ട്.

ഗവേഷണത്തിൽ പങ്കെടുക്കാൻ സ്വമേധയാ സമ്മതിക്കുന്നു.

ഘട്ടം 0/ഘട്ടം 1 /ഘട്ടം 2

പങ്കെടുക്കുന്ന വ്യക്തിയുടെ പേര്:

പങ്കെടുക്കുന്ന വ്യക്തിയുടെ ഒപ്പ് തീയതി:

എഴുതാനും വായിക്കാനും അറിയാത്ത വ്യക്തിയാണെങ്കിൽ ഒരു സാക്ഷി

ഈ പഠനത്തെ കുറിച്ചുള്ള വിവരങ്ങൾ മനസ്സിലാക്കി, ഈ പഠനത്തിൽ പങ്കെടുക്കാൻ ഈ വ്യക്തി സ്വമേധയാ സമ്മതിച്ചിട്ടുള്ളതാണ്

സാക്ഷിയുടെ പേര്:

സാക്ഷിയുടെ ഉപ്പ്, തീയതി :

ഇവിടെ കൈവിരൽ അടയാളം:

പങ്കെടുക്കുന്ന വ്യക്തിയുടെ പേര്:

ID:

എഴുതാനും വായിക്കാനും അറിയാത്ത വ്യക്തിയാണെങ്കിൽ ഒരു സാക്ഷി

ഈ പഠനത്തെ കുറിച്ചുള്ള വിവരങ്ങൾ മനസ്സിലാക്കി, ഈ പഠനത്തിൽ പങ്കെടുക്കാൻ ഈ വ്യക്തി സ്വമേധയാ സമ്മതിച്ചിട്ടുള്ളതാണ്

സാക്ഷിയുടെ പേര്:

സാക്ഷിയുടെ ഉപ്പ്, തീയതി :

ഇവിടെ കൈവിരൽ അടയാളം:

പങ്കെടുക്കുന്ന വ്യക്തിയുടെ പേര്:

ID:

Consent for Observation of animal suspected/suffering from rabies, testing if necessary and data permission form

Unique ID : ______________________

Name of Owner/Reporter: Date:

Phone: Species: Type/Type:

Sex: Age: Colour/marks:

Taluk: Panchayat: Village:

Landmark and other address details:

I, the undersigned, hereby certify that I am the owner/keeper of the animal described above. I hereby grant full authority to Dr./Ms./Mr./Mr./Ms./Mr./Ms……………………………… I do not hold the organization responsible for any unexpected or untoward incident during this process. I authorize _______________________ to collect and archive any sample necessary for diagnosis, and to use the sample and data for scientific publication.

By signing this form, I permit Mission Rabies to handle the animal as they see fit, and I have the authority to enforce this consent.

Date: __________________

Signature of Owner or Reporter: ___________________

Signature of Veterinarian/Team Leader: _______________________

പേവിഷ ബാധ സംശയിക്കുന്ന/ അനുഭവിക്കുന്ന മൃഗത്തിന്റെ നിരീക്ഷണം , ആവാശ്യമെങ്കിൽ പരിശോധനക്കും സമ്മതവും ഡാറ്റ പെർമിഷൻ ഫോമും

അദ്വിതീയ ഐഡി: ________________

ഉടമ/റിപ്പോർട്ടറുടെ പേര്:		തീയതി:
ഫോൺ:	സ്പീഷീസ്:	ഇനം/തരം:
ലിംഗം:	പ്രായം:	നിറം/അടയാളങ്ങൾ:
താലൂക്ക്:	പഞ്ചായത്ത്:	ഗ്രാമം:
ലാൻഡ്മാർക്കും മറ്റ് വിലാസ വിശദാംശങ്ങളും:		

മുകളിൽ വിവരിച്ച മൃഗത്തിന്റെ ഉടമ/പരിപാലകൻ ഞാനാണെന്ന് താഴെ ഒപ്പിട്ടിരിക്കുന്ന ഞാൻ ഇതിനാൽ സാക്ഷ്യപ്പെടുത്തുന്നു. ____ ഓർഗനൈസേഷന്റെ, ഡോ/

മിസ്/ മിസ്റ്റർ ____ന്, അവരുടെ ജീവനക്കാർക്ക്,

രക്ഷാപ്രവർത്തനം, വെറ്ററിനറി ചികിത്സ, ശസ്ത്രക്രിയ, പുനരധിവസിപ്പിക്കൽ, മോചിപ്പിക്കൽ, നിരീക്ഷണം, ക്വാറന്റൈൻ അല്ലെങ്കിൽ ഓർഗനൈസേഷന് ഉചിതമെന്ന് തോന്നുന്ന ഏതുവിധേനയും ഈ മൃഗത്തിന്റെ ജീവിതം അവസാനിപ്പിക്കുന്നതിനുള്ള പരിപൂർണ്ണമായ അധികാരം, എന്നിവയ്ക്ക് ഞാൻ സമ്മതം നൽകുന്നു. ഈ പ്രക്രിയയ്ക്കിടെ അപ്രതീക്ഷിതമോ അനിഷ്ട സംഭവമോ ഉണ്ടായാൽ സംഘടനയെ ഞാൻ ഉത്തരവാദിയാക്കുന്നില്ല. രോഗനിർണയത്തിന് ആവശ്യമായ ഏതെങ്കിലും സാമ്പിൾ ശേഖരിക്കാനും ആർക്കൈവ് ചെയ്യാനും, ശാസ്ത്രീയ പ്രസിദ്ധീകരണത്തിനായി സാമ്പിളും ഡാറ്റയും ഉപയോഗിക്കാനും ഞാൻ ______________ ന് അനുമതി നൽകുന്നു.

ഈ ഫോമിൽ ഒപ്പിടുന്നതിലൂടെ, മൃഗത്തെ അവർക്ക് അനുയോജ്യമെന്ന് തോന്നുന്ന രീതിയിൽ കൈകാര്യം ചെയ്യാൻ മിഷൻ റാബിസിന് ഞാൻ അനുമതി നൽകുന്നു, ഈ സമ്മതം നടപ്പിലാക്കാൻ എനിക്ക് അധികാരമുണ്ട്.

തീയതി: ________________

ഉടമയുടെയോ റിപ്പോർട്ടറുടെയോ ഒപ്പ് : ________________

മൃഗഡോക്ടറുടെ/ടീം ലീഡറുടെ ഒപ്പ്: ________________

സമ്മതം വാങ്ങുന്ന ഗവേഷകൻ്റെ/വ്യക്തിയുടെ പ്രസ്താവന

പങ്കെടുക്കാൻ സാധ്യതയുള്ള വ്യക്തിക്ക് ഞാൻ വിവര ഷീറ്റ് കൃത്യമായി വായിച്ചു, കൂടാതെ ഇനിപ്പറയുന്നവ ചെയ്യുമെന്ന് പങ്കെടുക്കുന്നയാൾ മനസ്സിലാക്കുന്നുവെന്ന് ഉറപ്പാക്കുകയും ചെയ്തു:

1. പങ്കെടുക്കുന്നവരുമായി ബന്ധപ്പെട്ട വിവരങ്ങൾ ഒരു ചോദ്യാവലി ഉപയോഗിച്ച് ചോദ്യങ്ങൾ ചോദിച്ച് മെഡിക്കൽ രേഖകളിൽ നിന്ന് ശേഖരിക്കും

പഠനത്തെ കുറിച്ച് ചോദ്യങ്ങൾ ചോദിക്കാൻ പങ്കാളിയെ അനുവദിച്ചിട്ടുണ്ടെന്ന് ഞാൻ സ്ഥിരീകരിക്കുന്നു, പങ്കെടുക്കുന്നയാൾ ചോദിച്ച എല്ലാ ചോദ്യങ്ങൾക്കും കൃത്യമായും എൻ്റെ കഴിവിൻ്റെ പരമാവധിയിലും ഉത്തരം നൽകിയിട്ടുണ്ട്. സമ്മതം നൽകാൻ വ്യക്തിയെ നിർബന്ധിച്ചിട്ടില്ലെന്നും സമ്മതം സ്വതന്ത്രമായും സ്വമേധയാ നൽകിയിട്ടുണ്ടെന്നും ഞാൻ സ്ഥിരീകരിക്കുന്നു.

ഈ ഐസിഎഫിൻ്റെ ഒരു പകർപ്പ് പങ്കെടുക്കുന്നയാൾക്ക് നൽകിയിട്ടുണ്ട്.

സമ്മതം എടുക്കുന്ന ഗവേഷകൻ്റെ/വ്യക്തിയുടെ പേര്
അച്ചടിക്കുക______________________

ഗവേഷകൻ്റെ / സമ്മതം വാങ്ങുന്ന വ്യക്തിയുടെ ഒപ്പ്_________________________

തീയതി _________________________

ദിവസം/മാസം/വർഷം
