# Supplementary material for: Integrated bite case management within a One Health framework for rabies elimination at the primary care level in Kerala, India: an implementation research protocol using a stepped-wedge cluster randomised design
Source: BMJ Open. 2026 Sep 23;16(9):e117497. doi: 10.1136/bmjopen-2026-117497 (PMC13629920; doi:10.1136/bmjopen-2026-117497)
Supplement: Supplementary file 7 [file bmjopen-16-9-s007.pdf]

StaRI (Standards of Reporting Implementation Studies) checklist				
**Title and abstract**				**Reported on page**
Title	**1**	Identification as an implementation study, and description of the methodology in the title and/or keywords		1
Abstract	**2**	Identification as an implementation study, including a description of the implementation strategy to be tested, the evidence-based intervention being implemented, and defining the key implementation and health outcomes.		4
**Introduction**				
Introduction	**3**	Description of the problem, challenge or deficiency in healthcare or public health that the intervention being implemented aims to address.		5-7
Rationale	**4**	The scientific background and rationale for the implementation strategy (including any underpinning theory/framework/model, how it is expected to achieve its effects and any pilot work).	The scientific background and rationale for the intervention being implemented (including evidence about its effectiveness and how it is expected to achieve its effects).	5-7
Aims and objectives	**5**	The aims of the study, differentiating between implementation objectives and any intervention objectives.		7
**Methods: description**				
Design	**6**	The design and key features of the evaluation, (cross referencing to any appropriate methodology reporting standards) and any changes to study protocol, with reasons		8
Context	**7**	The context in which the intervention was implemented. (Consider social, economic, policy, healthcare, organisational barriers and facilitators that might influence implementation elsewhere).		9
Targeted 'sites'	**8**	The characteristics of the targeted 'site(s)' (e.g locations/personnel/resources etc.) for implementation and any eligibility criteria.	The population targeted by the intervention and any eligibility criteria.	9

Description	**9**		A description of the implementation strategy	A description of the intervention	10
Sub-groups	**10**		Any sub-groups recruited for additional research tasks, and/or nested studies are described		Rural, Urban and Tribal mentioned under settings in page 9
**Methods: evaluation**					
Outcomes	**11**		Defined pre-specified primary and other outcome(s) of the implementation strategy, and how they were assessed. Document any pre-determined targets	Defined pre-specified primary and other outcome(s) of the intervention (if assessed), and how they were assessed. Document any pre-determined targets	14,15 &16
Process evaluation	**12**		Process evaluation objectives and outcomes related to the mechanism by which the strategy is expected to work		8, 10, 20
Economic evaluation	**13**		Methods for resource use, costs, economic outcomes and analysis for the implementation strategy	Methods for resource use, costs, economic outcomes and analysis for the intervention	8, 16, 18
Sample size	**14**		Rationale for sample sizes (including sample size calculations, budgetary constraints, practical considerations, data saturation, as appropriate)		19,20
Analysis	**15**		Methods of analysis (with reasons for that choice)		15,16 &17
Sub-group analyses	**16**		Any a priori sub-group analyses (e.g. between different sites in a multicentre study, different clinical or demographic populations), and sub-groups recruited to specific nested research tasks		16
