# Supplementary material for: Integrated bite case management within a One Health framework for rabies elimination at the primary care level in Kerala, India: an implementation research protocol using a stepped-wedge cluster randomised design
Source: BMJ Open. 2026 Sep 23;16(9):e117497. doi: 10.1136/bmjopen-2026-117497 (PMC13629920; doi:10.1136/bmjopen-2026-117497)
Supplement: Supplementary file 8 [file bmjopen-2026-117497.reviewer_comments.pdf]

PEER REVIEW HISTORY

BMJ Open publishes all reviews undertaken for accepted manuscripts. Reviewers are asked to complete a checklist review form and are provided with free text boxes to elaborate on their assessment. These free text comments are reproduced below.

ARTICLE DETAILS

Title (Provisional)

Integrated Bite Case Management within a One Health Framework for Rabies Elimination at the Primary Care Level in Kerala, India: An Implementation Research Protocol Using a Stepped-Wedge Cluster Randomized Design Short title: Primary Care-Based Integrated Bite Case Management for Rabies in Kerala

Authors

Thajudeen Nujum, Zinia; Luka, Martha M; Raghunathan, Dhanya; Beegum, Mufeeda; S, Harikumar; Bajaj, Prapti; Chandrashekar, Balaji; K, Rajamohanan; Mathew, Thomas; Soman, Biju; Asaria, Miqdad; Karuppusami, Reka; Gibson, Andrew; R, Sreenath M; Jose, Arun; John, Rony Ray; S, Aparna; R, Pratiush P; Abraham, Swapna Susan; B, Narayanan S; Chungath, Ummuselma; Indu, P; U, Anuja; S, Remadevi; Vijayakumar, Krishnapillai; S, Aiswarya S; Hampson, Katie

---

VERSION 1 - REVIEW

**Reviewer**	**1**
**Name**	**Bharti, Omesh**
**Affiliation**	**State Institute of Health and Family Welfare, Health**
**Date**	**28-Apr-2026**
**COI**	**None**

1. The document need to take account of the previously vaccinated people or those who had pre- exposure prophylaxis and are exposed again especially for cost effectiveness of PEP.

2. In case of a report of some dog showing ALFA signs or a dog on biting spree need to be tracked down even if no one having been bitten by them have come to the selected clinics.

**Reviewer**	**2**
**Name**	**Bhattacharya, Sudip**
**Affiliation**	**AIIMS Deoghar, Community Medicine**
**Date**	**20-May-2026**

COI **None**

The protocol addresses a highly important and timely public health issue, and the One Health integration is one of the strongest aspects of the study. The manuscript is ambitious, policy-oriented, and demonstrates strong collaboration between the health, veterinary, and local governance sectors. The stepped-wedge cluster randomized design is scientifically appropriate for implementation research and adds methodological strength compared to many previous rabies surveillance studies. The inclusion of qualitative evaluation, RE-AIM framework, and cost-effectiveness modelling makes the protocol comprehensive and potentially very impactful for rabies elimination programs in India. The manuscript also shows good contextual understanding of Kerala's health system and provides strong operational details regarding IBCM implementation.

However, despite these strengths, the protocol remains excessively lengthy and at several places becomes difficult to follow because of over-description of operational details. Many sections repeat similar concepts related to One Health integration, stakeholder coordination, and implementation processes. The manuscript would benefit from tightening of language and reducing repetitive explanations, especially in the introduction and intervention sections. Some sentences are very long and complicated, which affects readability. There are also occasional grammatical and formatting inconsistencies throughout the document.

The study design is strong overall, but some methodological concerns remain. The recruitment strategy of selecting only the "first two dog bite patients" presenting daily at PHCs may introduce selection bias and reduce representativeness. The rationale for this specific recruitment approach is not adequately justified. Similarly, although the stepped-wedge design is appropriate, there is limited discussion on how temporal confounding and secular trends will be controlled during analysis. The protocol mentions GLMM analysis, but more operational statistical clarity would improve confidence in the analysis plan.

The intervention itself is operationally intensive and depends heavily on coordination between multiple stakeholders, WhatsApp communication, hotline functioning, volunteers, NGOs, veterinary services, and local governance systems. While this reflects real-world implementation, the protocol does not sufficiently discuss risks related to inconsistent implementation across clusters. Monitoring of intervention fidelity needs more clarity. Dependence on phone follow-up may additionally lead to loss to follow-up among poorer or migratory populations.

The sample size section is detailed but somewhat confusing. Multiple calculations are presented for different components of the study, making it difficult for readers to clearly identify the final analytical sample relevant to the primary outcomes. Simplifying this section and clearly separating the RCT sample, baseline survey sample, and qualitative sample would improve clarity considerably.

The qualitative component is valuable, but the protocol appears very ambitious in scope. Conducting large numbers of IDIs, FGDs, field investigations, surveillance activities, ring vaccination, cost-effectiveness analysis, and implementation evaluation simultaneously may create operational burden. More discussion regarding feasibility, human resource management, and quality assurance mechanisms would strengthen the protocol further.

Another limitation is that the protocol acknowledges resource constraints and non-continuous monitoring, but the possible impact of these limitations on surveillance sensitivity and intervention effectiveness is not adequately discussed. This should be more transparently addressed in the limitations section.

Overall, this is a scientifically important and potentially high-impact implementation research protocol with strong translational value for rabies elimination in India. However, the manuscript would substantially improve with better language editing, reduction of repetitive content, simplification of methodological explanations, clearer justification of sampling procedures, and stronger discussion of operational feasibility and implementation variability.

VERSION 1 - AUTHOR RESPONSE

**Reviewer: 1**<br>**Dr. Omesh Bharti, State Institute of Health and Family Welfare**		
1.		
	The document need to take account of the previously vaccinated people or those who had pre- exposure prophylaxis and are exposed again especially for cost effectiveness of PEP.	Thank you for the suggestion – Re-exposure is already part of the trigger event since any dog bite is included which requires PEP. For the cost-effectiveness analysis (CEA) we will account for re-exposure. We will be looking at the CE of adding IBCM to the existing program. Since PrEP is not part of the existing program, this is beyond the scope of the present study, but we have examined PrEP in other work.
2.	In case of a report of some1. dog showing ALFA signs or a dog on biting spree need to be tracked down even if no one having been bitten by them have come to the selected clinics.	Yes – this is important and it forms one of the criteria for an event to be reported from the community – community trigger event – lines 323
**Reviewer: 2**<br>**Dr. Sudip Bhattacharya, AIIMS Deoghar**		
1.	The protocol addresses a highly important and timely public health issue, and the One Health integration is one of the strongest	Thanks for the very positive remarks

	aspects of the study. The manuscript is ambitious, policy-oriented, and demonstrates strong collaboration between the health, veterinary, and local governance sectors. The stepped-wedge cluster randomized design is scientifically appropriate for implementation research and adds methodological strength compared to many previous rabies surveillance studies. The inclusion of qualitative evaluation, RE-AIM framework, and cost-effectiveness modelling makes the protocol comprehensive and potentially very impactful for rabies elimination programs in India. The manuscript also shows good contextual understanding of Kerala's health system and provides strong operational details regarding IBCM implementation.	
2.	The protocol remains excessively lengthy and at several places becomes difficult to follow because of over-description of operational details. Many sections repeat similar concepts related to One Health integration, stakeholder coordination, and implementation processes. The manuscript would benefit from tightening of language and reducing repetitive explanations, especially in the introduction and intervention sections. Some sentences are very long and complicated, which affects readability.	We have now edited to avoid lengthy sentences and repetitions so as to improve readability.
3.	There are also occasional grammatical and formatting inconsistencies throughout the document.	Now corrected.
4.	The study design is strong overall, but some methodological concerns remain. The recruitment strategy of selecting only the "first two dog bite	This is an important observation.<br>We have added the following paragraph to the discussion: "A consecutive approach enrolling the first two eligible patients each

	patients" presenting daily at PHCs may introduce selection bias and reduce representativeness. The rationale for this specific recruitment approach is not adequately justified.	day was adopted for detailed follow-up, due to operational and human resource constraints in busy primary care settings. This strategy ensured feasibility within routine clinic workflows, minimized disruption to service delivery, and provided a standardized and consistent recruitment procedure across sites. While random selection of patients would have been methodologically preferable, it was not operationally feasible in real-time clinical settings and could have delayed case capture and follow-up procedures. The chosen approach reduced investigator-driven convenience selection, although it does not eliminate selection bias. Importantly, hospital- and community-triggered reporting of rabies events ensured that surveillance and response to probable and confirmed cases were not restricted to enrolled participants."<br><br>In addition we added this sentence added to methods: "To assess the reach and coverage of the surveillance system, the total number of dog-bite patients presenting to each participating PHC during the study period will be recorded and reported alongside the number enrolled for detailed follow-up." Lines 255-257
5.	However, despite these strengths, although the stepped-wedge design is appropriate, there is limited discussion on how temporal confounding and secular trends will be controlled during analysis.	Added this sentence: "A generalized linear mixed model (GLMM) will be used for analysis, with fixed effects for intervention exposure and time periods to account for secular trends inherent in the stepped-wedge cluster design. The model will include random effects for clusters (PHCs) to account for within-cluster correlation. This structure enables estimation of intervention effects while appropriately adjusting for both clustering and temporal confounding", Lines 491-496
6.	The protocol mentions GLMM analysis, but more operational statistical clarity would improve	We have tried to improve the clarity with edits to lines 491-496

	confidence in the analysis plan.	
7.	The intervention itself is operationally intensive and depends heavily on coordination between multiple stakeholders, WhatsApp communication, hotline functioning, volunteers, NGOs, veterinary services, and local governance systems. While this reflects real-world implementation, the protocol does not sufficiently discuss risks related to inconsistent implementation across clusters. Monitoring of intervention fidelity needs more clarity.	We thank the reviewer for highlighting the importance of implementation fidelity in this multi-component intervention. We acknowledge that the intervention is operationally intensive and depends on coordination across multiple stakeholders, including health services, veterinary services, NGOs, volunteers, and local governance systems, which may lead to varied implementation across clusters.<br>To address this, the study includes a structured implementation fidelity monitoring plan. Core intervention components (WhatsApp communication pathways, hotline functionality, referral linkages, and response coordination) will be tracked using predefined process indicators across clusters and over time. Routine monitoring will document whether each component is functional and actively used, and implementation performance will be reviewed periodically to identify gaps and ensure corrective actions where required. This will allow assessment of both intervention effectiveness and consistency of delivery across study sites. Lines 472-478
8.	Dependence on phone follow-up may additionally lead to loss to follow-up among poorer or migratory populations.	Agreed. However, this dependency occurs during both the control and intervention periods, and is not expected to affect the effectiveness of the intervention. Besides we include the reporting of community trigger events to additionally capture events from poorer and migratory populations, since this reporting is done by health workers, ASHA, or any lay person in the community
9.	The sample size section is detailed but somewhat confusing. Multiple calculations are presented for different components of the study, making it difficult for readers to clearly identify the final analytical sample relevant to the primary outcomes. Simplifying this section	Edited this section to be clearer with three paragraphs: 1) for the cross-sectional study; 2) for the RCT and 3) for the Qualitative study.

	and clearly separating the RCT sample, baseline survey sample, and qualitative sample would improve clarity considerably.	
10.	The qualitative component is valuable, but the protocol appears very ambitious in scope. Conducting large numbers of IDIs, FGDs, field investigations, surveillance activities, ring vaccination, cost-effectiveness analysis, and implementation evaluation simultaneously may create operational burden. More discussion regarding feasibility, human resource management, and quality assurance mechanisms would strengthen the protocol further.	Yes this is challenging, but based on previous experience we were confident to propose this, given the valuable collaboration of the multiple partners we have engaged. We have included a paragraph to address this, Lines 686-695
11.	Another limitation is that the protocol acknowledges resource constraints and non-continuous monitoring, but the possible impact of these limitations on surveillance sensitivity and intervention effectiveness is not adequately discussed. This should be more transparently addressed in the limitations section.	Edited to address this, lines 699-701
12.	Overall, this is a scientifically important and potentially high-impact implementation research protocol with strong translational value for rabies elimination in India. However, the manuscript would substantially improve with better language editing, reduction of repetitive content, simplification of methodological explanations, clearer justification of sampling procedures, and stronger discussion of operational feasibility and implementation variability.	Thanks – we have tried to correct and modify as per the suggestions as much as we could.
